# The Efficacy and Safety of Adding Teniposide to Temozolomide Plus Bevacizumab in Recurrent Grade 4 Glioma: A Real-World Exploratory Single-Center Study

**DOI:** 10.3390/jcm15207827

**Published:** 2026-10-10

**Authors:** Zekun Deng, Hongqing Cai, Yujia Chen, Qi Liu, Huaixu Li, Cheng Fang, Ming Yang, Haipeng Qian, Jinghai Wan

**Affiliations:** Department of Neurosurgery, National Cancer Center/National Clinical Research Center for Cancer/Cancer Hospital, Chinese Academy of Medical Sciences and Peking Union Medical College, Beijing 100021, China; zekundeng@163.com (Z.D.);

**Keywords:** recurrent grade 4 glioma, teniposide, temozolomide, bevacizumab, survival

## Abstract

**Background:** Salvage options for recurrent grade 4 glioma (GBM) remain limited and no single standard of care has been established after recurrence. Teniposide (VM-26) has been reintroduced into clinical practice, but its benefit when added to temozolomide (TMZ) plus bevacizumab (Bev) in recurrent grade 4 glioma remains unclear. **Methods:** This single-center, real-world exploratory study retrospectively enrolled 53 patients with recurrent grade 4 glioma who received either teniposide plus TMZ and Bev (VM26 + TMZ + Bev, *n* = 26) or TMZ plus Bev (TMZ + Bev, *n* = 27). The control group combined patients treated before teniposide became available again in China (2020–2022) with those treated concurrently with the triplet (2023–2026). Safety was assessed using the Common Terminology Criteria for Adverse Events (CTCAE), version 5.0; tumor response was evaluated according to the Response Assessment in Neuro-Oncology (RANO) criteria on an intention-to-treat basis, and survival was estimated by the Kaplan–Meier method. **Results:** Median progression-free survival (PFS) was 7.8 months (95% confidence interval (CI), 4.9–10.9) in the VM26 + TMZ + Bev group versus 5.1 months (95% CI, 4.4–6.8) in the TMZ + Bev group (log-rank *p* = 0.037; unadjusted hazard ratio (HR) 0.50, 95% CI, 0.25–0.97), and median overall survival (OS) was 12.7 months (95% CI, 8.4–15.4) versus 8.5 months (95% CI, 7.3–9.7) (log-rank *p* = 0.042; unadjusted HR 0.47, 95% CI, 0.23–0.98). Multivariable adjustment strengthened these estimates, whereas propensity score weighting and matching gave estimates of the same direction that did not reach significance. The objective response rate (ORR) was 26.9% (7/26) versus 14.8% (4/27) (*p* = 0.327) and the disease control rate (DCR) was 80.8% versus 77.8% (*p* = 1.000). Grade ≥ 3 adverse events (AEs) occurred in 11.5% versus 7.4% of patients (*p* = 0.669); leukopenia and neutropenia were more frequent with teniposide (26.9% vs. 3.7%; *p* = 0.024) but lost significance after Benjamini–Hochberg correction (q = 0.122). No unexpected toxicity types were observed, although any-grade leukopenia and neutropenia were more frequent with teniposide and should be anticipated in clinical use. **Conclusions:** In this exploratory real-world cohort, adding teniposide to TMZ plus Bev was associated with longer PFS and OS and a numerically higher ORR, without a significant increase in grade ≥ 3 toxicity, although any-grade leukopenia and neutropenia were more frequent and should be anticipated; however, treatment era could not be fully separated from treatment effect: in an era-matched analysis restricted to the concurrently treated cohorts, the direction of the effect was preserved but did not reach significance. The survival differences rested on a small number of events and were not robust to reclassification of individual patient outcomes. These findings warrant confirmation in prospective randomized studies.

## 1. Introduction

Glioblastoma (GBM), the most aggressive adult primary brain tumor classified as grade 4 by the World Health Organization (WHO) classification of central nervous system (CNS) tumors [1], accounts for approximately 14.5% of all primary CNS tumors and 48% of malignant CNS tumors worldwide [2]. Typically diagnosed at a median age of 64 years with a male predominance (male-to-female ratio 1.6:1), GBM exhibits a marked increase after age 50 [2]. The current standard therapy for GBM comprises maximal safe resection, concurrent temozolomide (TMZ)-based chemoradiotherapy, and adjuvant TMZ, supplemented by tumor-treating fields [3]. Despite multimodal therapy, the median overall survival (OS) of GBM patients remains dismal, with median OS of about 20 months and a 5-year survival rate below 10% [4]. 

Chemotherapy serves as a cornerstone of multimodal GBM management, primarily acting via direct tumor cell cytotoxicity, inhibition of deoxyribonucleic acid (DNA) synthesis, or induction of apoptosis to retard disease progression. GBM chemotherapy centers on TMZ, which is hampered by notable limitations: approximately 50% of patients exhibit primary resistance, with nearly all ultimately experiencing disease recurrence [5]. Additional constraints include O6-methylguanine-DNA methyltransferase (MGMT) promoter methylation-dependent efficacy [6] and myelosuppression [4]. Consequently, exploration of novel therapeutic agents and strategies represents a critical approach to overcome the therapeutic impasse in GBM. 

In recent years, clinical trials targeting GBM have made incremental progress yet mostly ended in failure. Regarding immunotherapy, despite initial promise, candidates including nivolumab, the epidermal growth factor receptor variant III (EGFRvIII)-targeted peptide vaccine rindopepimut, and recombinant poliovirus PVSRIPO failed to maintain efficacy or achieve clinical endpoints in subsequent trials [7]. Vorasidenib, a mutant isocitrate dehydrogenase 1/2 (IDH1/IDH2) enzyme inhibitor, significantly improved progression-free survival (PFS) in Grade 2 IDH-mutant glioma patients, but its efficacy in high-grade gliomas remains undetermined [8]. Tyrosine kinase inhibitors (TKIs) targeting the epidermal growth factor receptor (EGFR) pathway largely failed to show meaningful efficacy, with inconsistent evidence of target engagement [7]. To date, agents targeting the phosphatidylinositol 3-kinase (PI3K)/mechanistic target of rapamycin (mTOR) pathway have also demonstrated limited utility: the pan-PI3K TKI buparlisib showed minimal single-agent activity in recurrent PI3K-activated GBM [9], and mTOR inhibitors such as everolimus and temsirolimus similarly failed in phase 2 trials [10,11]. Anti-angiogenic therapies such as bevacizumab (Bev) improved PFS without extending OS in GBM [7]. Additionally, cediranib failed to prolong PFS or enhance survival in recurrent GBM in a randomized trial [12].

Teniposide (VM-26), a semi-synthetic podophyllotoxin derivative, functions as a topoisomerase II (Topo II) inhibitor. Its antitumor activity is mediated by selective binding to the Topo II-DNA complex, stabilizing enzyme-induced DNA double-strand breaks to inhibit DNA repair/replication and induce tumor cell apoptosis [13]. Compared to conventional Topo II inhibitors such as etoposide, VM-26 exhibits greater lipophilicity, and its penetration into human intracerebral tumors has been demonstrated directly, conferring a distribution advantage in central nervous system tumors [14]. In glioma therapy, early studies summarized in a review of chemotherapy for adult gliomas reported a modest 28% response rate for single-agent VM-26 in recurrent gliomas, with evidence of synergy when it was combined with other agents [15]. A subsequent phase 2 study reported a 6-month PFS rate of 34.8% using a VM-26–carboplatin combination in patients with recurrent oligodendroglioma or mixed oligoastrocytoma [16]. 

Despite its potential in brain tumor therapy, VM-26’s clinical application is constrained by several factors: first, its limited single-agent efficacy necessitates combination strategies; second, existing regimens predominantly focus on recurrent tumors, and standardized protocols for newly diagnosed GBM are still lacking. More critically, VM-26 underwent a global production suspension in recent years, resulting in drug shortages that substantially hindered clinical research progression. Its reintroduction in China in 2022 has alleviated supply constraints and revitalized opportunities for comprehensive clinical exploration.

This study aims to address the limited understanding of VM-26-based therapy through an analysis of clinical data from patients with recurrent grade 4 glioma receiving this therapy, using patients treated with TMZ plus bevacizumab without teniposide as the comparator. The objective is to evaluate its safety and efficacy and to assess the extent to which the observed differences may be attributable to the treatment era, thereby providing novel evidence-based insights for the management of recurrent grade 4 glioma.

## 2. Materials and Methods

### 2.1. Study Design and Setting 

This was a single-center, retrospective, non-interventional exploratory cohort study conducted in the Department of Neurosurgery, National Cancer Center/National Clinical Research Center for Cancer/Cancer Hospital, Chinese Academy of Medical Sciences and Peking Union Medical College (Beijing, China). All consecutive adult patients with recurrent grade 4 glioma who started bevacizumab-based salvage chemotherapy at our center were screened. The enrollment period ran from 2019 to 2026, and follow-up was updated to 31 August 2026, which served as the data cut-off date. Enrollment began in 2019, whereas the date on which chemotherapy was first started after recurrence—the time origin for all efficacy and survival analyses—ranged from 13 February 2020 to 3 April 2026 in this cohort. The study was conducted in accordance with the ethical principles of the Declaration of Helsinki and was reviewed and approved by the Ethics Committee of Cancer Hospital, Chinese Academy of Medical Sciences (approval number 2024080716300902; approval date: 23 August 2024). The approval covered the conduct of the study and the access to and use of clinical data from patients who had provided informed consent for scientific research and publication; each patient treated during the study period signed the relevant informed consent form at the initiation of treatment. Reporting follows the STROBE recommendations for observational studies. Because the study describes routine clinical practice, treatment allocation was not randomized, and the study was not designed or powered to test a formal superiority hypothesis; all analyses are therefore exploratory in nature.

### 2.2. Participants

Patients were eligible if they (i) were aged 18 years or older; and (ii) had a histologically confirmed diagnosis of diffuse glioma, WHO grade 4, according to the fifth edition of the WHO Classification of Tumors of the Central Nervous System (WHO CNS5, 2021), comprising glioblastoma, IDH-wildtype, WHO grade 4; astrocytoma, IDH-mutant, WHO grade 4; or glioblastoma, WHO grade 4 (NOS). Eligible patients also had to have (iii) radiological or clinical evidence of recurrence or progression after initial standard treatment, which consisted of surgery followed by radiotherapy with or without concomitant and adjuvant temozolomide according to the standard of care at the time; and (iv) a Karnofsky Performance Status (KPS) score of at least 60 with an expected survival of at least 3 months. Finally, patients had to be (v) scheduled to receive either teniposide plus temozolomide plus bevacizumab (VM26 + TMZ + Bev) or temozolomide plus bevacizumab (TMZ + Bev) as salvage chemotherapy. Because the study intended to describe the outcome of a course of salvage treatment rather than of a single dose, patients had either to have completed at least two 28-day cycles or to have documented progression or death before completing two cycles. Five patients who started bevacizumab-based salvage chemotherapy during the study period but discontinued it before completing two cycles, without documented progression or death, were therefore not eligible for inclusion; the analysis set comprised the 53 patients who met all eligibility criteria. Patients also had to have (vi) complete baseline evaluation and follow-up records, including complete blood count, serum biochemistry, thyroid function, immune function and imaging studies performed before treatment initiation. The number of radiotherapy fractions and adjuvant temozolomide cycles received before recurrence was recorded; radiotherapy fractions were available only for patients who received radiotherapy as part of their initial treatment. Patients were excluded if they had a second primary malignancy; severe cardiopulmonary, hepatic or renal dysfunction; an acute cerebrovascular event; a severe autoimmune disorder or other systemic comorbidity precluding treatment tolerance; or incomplete follow-up data with loss to follow-up.

### 2.3. Treatment Eras and Comparison Groups

Teniposide became available again in China only after 2022. Patients treated at our center before the end of 2022 therefore had no access to the teniposide-containing regimen, whereas from 2023 onwards both regimens were available and patients were treated concurrently. Patients were accordingly classified by treatment era and regimen into three groups: those treated with TMZ + Bev during the non-overlap period (2020–2022; *n* = 12), those treated with TMZ + Bev during the overlap period (2023–2026; *n* = 15), and those treated with VM26 + TMZ + Bev during the overlap period (2023–2026; *n* = 26). The two TMZ + Bev cohorts were additionally pooled into a single control group spanning both periods (*n* = 27).

Three comparisons were pre-specified. The primary analysis compared the VM26-containing triplet with the pooled TMZ + Bev control group. An era-matched analysis restricted to the two overlap-period cohorts compared the triplet with the concurrent TMZ + Bev cohort; because both groups were treated during the same calendar period, this contrast isolates the effect of adding teniposide without confounding by treatment era. The same TMZ + Bev regimen was also compared across the two periods in order to quantify the secular change in outcome over time. During the overlap period the choice between the two regimens was made by the treating neuro-oncologist on the basis of clinical considerations, which constitutes confounding by indication and was addressed by the pre-specified adjusted, propensity score and era-matched analyses described in Section 2.8.

The considerations recorded in the medical record at the time of treatment selection were abstracted for all 53 patients and are listed for each patient in Table S9. Teniposide was added preferentially for patients whose disease was considered more difficult to control—a tumor judged difficult to resect (12 of 26 patients [46.2%] versus 6 of 27 [22.2%]), multiple lesions at recurrence (10 [38.5%] versus 6 [22.2%]), radiation necrosis (3 versus 1) or progression despite prior therapy (13 versus 15)—and for patients with MGMT promoter methylation (8 [30.8%] versus 5 [18.5%]), a marker of greater responsiveness to alkylating chemotherapy. A good performance status was equally common in the two groups (24 versus 24). Several of these considerations, and multiple lesions at recurrence in particular, are also prognostic factors; treatment allocation was therefore not independent of prognosis, and the resulting confounding by indication was addressed analytically, as described in Section 2.8.

### 2.4. Molecular Testing

Molecular profiling was performed on formalin-fixed, paraffin-embedded tumor tissue obtained at the initial operation, using a targeted next-generation sequencing panel covering IDH1 and IDH2 mutations, the TERT promoter, MGMT promoter methylation, CDKN2A/B copy number, 1p/19q status and EGFR amplification, together with the copy-number status of chromosomes 7 and 10. Testing was performed in the Department of Pathology of our institution according to standard protocols. Results were reported as mutant or wildtype for IDH1/2 and TERT, methylated or unmethylated for the MGMT promoter, and present or absent for CDKN2A/B homozygous deletion, 1p/19q codeletion, EGFR amplification, +7 and −10. Molecular testing was performed in 39 patients (73.6%); in the remaining 14 patients (26.4%), archival tissue was unavailable or testing had not been requested, and these patients are reported as “Not tested” in Table 1. No 1p/19q codeletion was detected in any tested patient.

### 2.5. Treatment Regimens

Salvage chemotherapy was administered in 28-day cycles. The triplet regimen consisted of teniposide 50 mg/m^2^ intravenously on days 1–3, temozolomide 150 mg/m^2^ orally on days 1–5, and bevacizumab 10 mg/kg intravenously on day 1 of each cycle. The doublet regimen consisted of temozolomide 150 mg/m^2^ on days 1–5 and bevacizumab 10 mg/kg on day 1, given on the same 28-day schedule. Bevacizumab was administered every four weeks so that it could be given on the same day as the cytotoxic chemotherapy; this 4-weekly schedule is the established practice for recurrent glioblastoma at our institution and differs from the 2- or 3-weekly regimens used in some prospective trials. Treatment was continued until disease progression, unacceptable toxicity or withdrawal of consent. Dose reductions were permitted for hematological toxicity, with teniposide reduced from 50 to 40 mg/m^2^ and temozolomide reduced from 150 to 100 mg/m^2^ per day. During salvage therapy, dose reduction was required in 5 patients (teniposide, 3 patients; temozolomide, 2 patients), and treatment was discontinued because of an adverse event in 4 patients. Corticosteroids were administered at the discretion of the treating physician, and the dose and duration of dexamethasone treatment during salvage therapy were recorded for each patient. Granulocyte colony-stimulating factor, antiemetics and antiepileptic drugs were given as clinically indicated. Treatments received after progression were recorded for every patient.

### 2.6. Assessments and Outcome Definitions

#### 2.6.1. Radiological Assessment

Contrast-enhanced magnetic resonance imaging (MRI), including T1-weighted post-gadolinium and T2-weighted or fluid-attenuated inversion recovery sequences, was performed at baseline and every two to three treatment cycles, that is approximately every 8 to 12 weeks, until progression. The date of first administration of salvage chemotherapy was defined as the index date, and the interval from the index date to the first post-baseline response assessment was recorded for every patient; this interval was compared between groups in order to verify that the groups were assessed within comparable time windows. Tumor response was evaluated according to the Response Assessment in Neuro-Oncology (RANO) criteria (version 2.0, 2023) and classified as complete response, partial response, stable disease or progressive disease. As the study was retrospective and based on routine care, imaging was reviewed locally by the treating neuro-oncologists together with radiologists at our institution; no central review and no blinding of the assessors was performed, which is acknowledged as a limitation. All efficacy analyses were performed on an intention-to-treat basis: patients without a documented assessment were retained in the denominator, and progression or death occurring before the assessment window was counted as progressive disease. The objective response rate (ORR; complete or partial response) and the disease control rate (DCR; complete response, partial response or stable disease) were calculated for each group, and early progression, defined as progression within 2 months of the index date, was recorded.

#### 2.6.2. Survival Endpoints

Progression-free survival (PFS) was defined as the time from the index date to documented progression or death from any cause, whichever occurred first. Overall survival (OS) was defined as the time from the index date to death from any cause. Patients who were alive without progression were censored at the date of last follow-up, and patients who were alive at the data cut-off were censored on 31 August 2026. Survival times were calculated in days and converted to months by dividing by 30.4375. Follow-up consisted of scheduled outpatient visits with clinical assessment and MRI; patients who did not attend scheduled imaging were contacted by telephone in order to ascertain survival status.

#### 2.6.3. Safety Assessment

Adverse events were graded according to the National Cancer Institute Common Terminology Criteria for Adverse Events (CTCAE), version 5.0 (2017). Because the study was retrospective, adverse events were identified by reviewing the medical records—laboratory results, imaging reports, outpatient and inpatient notes, and medication records—for every cycle of salvage therapy; routine hematological and biochemical tests were performed before each cycle. The worst grade recorded for each patient during salvage therapy was used for analysis, and a grade of 0 indicated that the event did not occur. Hematological and non-hematological events were summarized separately, and dose reduction and discontinuation of treatment owing to an adverse event were recorded as described above.

### 2.7. Data Collection and Quality Control

Data were extracted from the institutional electronic medical record system and the picture archiving and communication system. A predefined variable dictionary specified the definition, source and coding of every variable, including demographic and baseline characteristics, previous treatment, molecular results, treatment dates and doses, dose modifications, response assessments, adverse events and survival status. All records were extracted independently by two investigators, and discrepancies were resolved by consensus with a third senior investigator. Patient identifiers were removed before analysis. Patient-level data and the ten supplementary tables containing the pre-specified analyses and the additional analyses requested during review (Tables S1–S10), including the analyses accounting for treatment era and the analysis restricted to glioblastoma, IDH-wildtype, are provided as Supplementary Materials.

### 2.8. Statistical Analysis

#### 2.8.1. Descriptive and Comparative Analyses

Continuous variables were tested for normality with the Shapiro–Wilk test and for equality of variances with Levene’s test. Variables that were normally distributed in all compared groups were compared with Student’s *t*-test, with Welch’s correction when variances were unequal; otherwise, the Mann–Whitney U test was used. Categorical variables were compared with the Pearson χ^2^ test or, for 2 × 2 tables with any expected cell count below 5, with the Fisher exact test; for the pooled control efficacy comparisons the Fisher exact test was applied to all 2 × 2 tables so that they were consistent with the primary efficacy analysis; for r × c tables with any expected cell count below 5, a Monte-Carlo exact test with 20,000 replicates was used. Baseline characteristics are reported descriptively together with the corresponding *p* values, and no adjustment for multiplicity was applied to these comparisons. For the main between-group comparisons, differences are expressed as the difference in medians for continuous variables and as the difference in percentage points for categorical variables. All efficacy and survival analyses were performed on the full analysis set (*n* = 53) with the index date as the time origin; the number of cycles of salvage chemotherapy completed was not used to select patients for the survival analyses, and patients who progressed or died during the first two cycles were retained and counted as events (the earliest recorded progression occurred on day 34 and the earliest death on day 40). The number of cycles of salvage chemotherapy received is reported in Table 1.

#### 2.8.2. Survival Analysis

PFS and OS were estimated by using the Kaplan–Meier method and compared with the two-sided log-rank test. Hazard ratios (HRs) with 95% confidence intervals (CIs) were obtained from Cox proportional hazards models, both univariable and multivariable. The 95% CIs of the median survival times were derived from the Greenwood variance estimator with a log–log transformation. The proportional hazards assumption was assessed with scaled Schoenfeld residuals (Table S3); the treatment effect, which is the estimate of primary interest, satisfied the assumption for both endpoints (*p* = 0.173 for PFS and *p* = 0.293 for OS), whereas KPS ≥ 90 showed a borderline violation in the PFS model (*p* = 0.030). Five pre-specified approaches were therefore applied and are reported side by side in Table S4: the primary model with KPS as a covariate; a Cox model stratified by KPS; a time-varying coefficient model including a KPS × ln(t) interaction term, with ln(t) centered on its median to avoid structural collinearity between the main effect and the interaction term; and landmark analyses at 60 and 90 days.

#### 2.8.3. Confounding Control and Treatment Era

The multivariable Cox models included the treatment group and three covariates selected a priori: KPS ≥ 90, glioblastoma with IDH-wildtype status (WHO CNS5 grade 4), and completion of re-resection at recurrence before the start of salvage chemotherapy. Multicollinearity among the candidate covariates was examined with variance inflation factors and pairwise correlation coefficients (Table S7). Because MGMT promoter methylation, TERT promoter mutation and CDKN2A/B homozygous deletion were moderately collinear with one another (|r| = 0.79–0.86), the primary model used the integrated diagnosis (glioblastoma, IDH-wildtype) rather than individual molecular markers; MGMT promoter methylation was examined separately in a sensitivity analysis (Table S5). Three pre-specified strategies were used to address confounding by treatment era and by indication. First, the era-matched comparison restricted to the two overlap-period cohorts (Section 2.3). Second, a propensity score analysis: the probability of receiving the teniposide-containing triplet was estimated by logistic regression including age, sex, IDH-wildtype status, number of lesions at recurrence, re-resection at recurrence, extent of resection at initial surgery, KPS ≥ 90 and comorbidity burden (McFadden pseudo-R^2^ = 0.233; Table S2). The resulting score was used as a single covariate in a Cox model, as the basis of stabilized inverse-probability-of-treatment weights, and as the basis of 1:1 nearest-neighbor matching with a caliper of 0.2 × the standard deviation of the logit of the propensity score, which yielded 13 matched pairs analyzed by stratified Cox regression. Third, the cross-era comparison of the same TMZ + Bev regimen, which quantifies the secular trend in outcome and thereby indicates the direction of the bias that a comparison with a historical control would introduce. The results of these three strategies are reported in Table S8. Furthermore, because multiple lesions at recurrence were numerically more frequent in the triplet group and were raised as a possible source of selection bias, the primary multivariable model was refitted with this variable added as a fourth covariate (Table S1). In addition, and in response to peer review, the survival analyses were repeated in the molecularly defined subset of patients with glioblastoma, IDH-wildtype, WHO grade 4 (Table S10).

#### 2.8.4. Pre-Specified Sensitivity Analyses

Eleven analysis strategies were specified before the analyses were performed and are reported in full in Table S1: univariable Cox regression; the multivariable Cox model (primary model); the propensity score as a single covariate; inverse probability of treatment weighting; propensity score matching with stratified Cox regression; and a Cox model stratified by KPS. The remaining pre-specified strategies were a time-varying coefficient model; landmark analyses at 60 and 90 days; a complete-case analysis restricted to the 39 patients who underwent molecular testing; and a complete-case analysis with additional adjustment for MGMT promoter methylation. In addition, the baseline characteristics of tested and untested patients were compared using standardized mean differences in order to assess potential selection bias (Table S6).

#### 2.8.5. Multiplicity

The PFS and OS analyses were each based on a single pre-specified comparison and were therefore not adjusted for multiple comparisons. The eleven strategies reported in Table S1 are exploratory and are presented without adjustment, and their consistency rather than any individual *p* value should be interpreted. Baseline comparisons were likewise not adjusted. For the safety analysis, multiplicity across the 10 individual adverse events was addressed with the Benjamini–Hochberg procedure, and Benjamini–Hochberg-adjusted q values are reported alongside the unadjusted *p* values. Composite endpoints (any adverse event, any grade ≥ 3 adverse event, any hematological adverse event and any non-hematological adverse event) were pre-specified as single endpoints and were therefore not included in the Benjamini–Hochberg family.

#### 2.8.6. Missing Data

No imputation was performed. Molecular testing was not carried out in 14 of the 53 patients (26.4%) because archival tissue was unavailable or testing had not been requested; this represents structural, rather than random, missingness. Patients who were not tested cannot be assigned an IDH status, and this limitation is addressed by the pre-specified complete-case analysis restricted to the 39 tested patients (Tables S1 and S5), in which the number of events decreased from 38 to 28 for PFS and from 35 to 25 for OS, corresponding to a reduction in events per variable from 9.5 to 7.0 and from 8.8 to 6.3, respectively. All other variables were complete.

#### 2.8.7. Sample Size, Software and Reporting

No a priori sample size calculation was performed, because the study was designed as an exploratory analysis of all consecutive eligible patients treated at a single center; 53 patients were included, with 38 PFS events and 35 OS events. The study was therefore not powered to detect moderate differences in the magnitude that would be clinically relevant, and the results should be regarded as hypothesis-generating. All analyses were performed with Python (version 3.13); Kaplan–Meier survival analysis, log-rank tests and Cox proportional hazards models were performed with the lifelines package (version 0.30.3), and between-group comparisons were performed with SciPy (version 1.18.1) and statsmodels (version 0.15.0). Two-sided *p* values below 0.05 were considered statistically significant, and exact *p* values are reported to three decimal places, except for values quoted from the cited literature, which are reproduced as published. Hazard ratios with 95% confidence intervals are reported for all time-to-event analyses.

## 3. Results

### 3.1. Study Population and Baseline Characteristics

The study population comprised 53 patients with recurrent grade 4 glioma treated with a bevacizumab-based regimen. Patients were grouped by treatment era and regimen into 12 patients treated with TMZ plus bevacizumab during the non-overlap period (2020–2022), 15 treated with the same doublet during the overlap period (2023–2026) and 26 treated with the VM26 (teniposide) triplet during the overlap period (2023–2026); the two TMZ plus bevacizumab cohorts were additionally pooled into a single control group (*n* = 27) so that the two regimens could be compared directly. Baseline characteristics were broadly comparable across the groups, with one qualification: because molecular testing was not performed in a substantial proportion of patients (15.4% of the triplet group and 37.0% of the pooled control group), comparability for IDH, TERT, MGMT, CDKN2A/B and 1p/19q status can be assessed only in the patients who were tested; among the two concurrently treated cohorts, the testing rates were similar (84.6% vs. 80.0%; *p* = 0.693). The median age was 62.0 years in the pooled control group and 51.5 years in the VM26 triplet group (difference −10.5; *p* = 0.433), and 37.0% and 61.5% of patients, respectively, were men (+24.5 percentage points; *p* = 0.074). Body mass index, comorbidity burden, baseline KPS (median 90 in every group), extent of initial resection and the interval from initial treatment to recurrence (median 9.4 vs. 10.3 months; *p* = 0.471) did not differ significantly. Against the pooled control group, patients treated with the VM26 triplet had received markedly fewer adjuvant TMZ cycles (median 1.0 vs. 5.5; difference −4.5; *p* = 0.003), less often underwent surgery at recurrence (23.1% vs. 51.9%; −28.8 percentage points; *p* = 0.031), less often completed re-resection before the start of study chemotherapy (19.2% vs. 40.7%; −21.5 percentage points; *p* = 0.088) and more frequently had IDH-wildtype glioblastoma, WHO CNS5 grade 4 (76.9% vs. 48.1%; +28.8 percentage points; *p* = 0.031). Multiple recurrent tumors were likewise numerically more frequent in the VM26 triplet group (38.5% vs. 22.2%; *p* = 0.443), although this difference was not statistically significant. These pooled comparisons are not adjusted for treatment era. In the era-matched comparison restricted to the two overlap-period cohorts, neither re-resection at recurrence (−16.9 percentage points; *p* = 0.300), the proportion of IDH-wildtype glioblastoma (+16.9 percentage points; *p* = 0.300) nor the proportion with multiple recurrent tumors (38.5% vs. 26.7%; *p* = 0.512) differed significantly, whereas the lower prior adjuvant TMZ exposure in the VM26 triplet group persisted (−4.0 cycles; *p* = 0.025); the two TMZ plus bevacizumab cohorts were themselves comparable in prior treatment intensity (adjuvant TMZ cycles, median 5.5 vs. 5.0, *p* = 0.623; radiotherapy fractions, 30 vs. 29, *p* = 0.653), supporting their pooling. Molecular testing was performed in 84.6% of the VM26 triplet group versus 63.0% of the pooled control group (+21.6 percentage points; *p* = 0.074); no 1p/19q codeletion was detected. Thus, once treatment era was taken into account, the only substantial residual difference between the two regimens was the lower number of adjuvant TMZ cycles received before study treatment in the VM26 triplet group (Table 1).

### 3.2. Kaplan–Meier Survival Analysis

Kaplan–Meier analysis showed that adding teniposide to TMZ and bevacizumab was associated with longer survival (Table 2). The median progression-free survival (PFS) was 7.8 months (95% CI 4.9–10.9) in the VM26 + TMZ + Bev arm versus 5.1 months (95% CI 4.4–6.8) in the TMZ + Bev arm (log-rank χ^2^ = 4.358, *p* = 0.037), and the median overall survival (OS) was 12.7 months (95% CI 8.4–15.4) versus 8.5 months (95% CI 7.3–9.7) (log-rank χ^2^ = 4.147, *p* = 0.042). The separation between the curves widened beyond the medians: 12-month PFS was 24.4% (95% CI 6.8–47.6) versus 5.6% (95% CI 0.4–21.9), and 12-month OS was 50.7% (95% CI 26.9–70.4) versus 5.0% (95% CI 0.4–20.6). The OS benefit emerged only after approximately 6 months, at which point the rates were still similar and numerically higher in the control arm (79.1% vs. 85.0%). Events had occurred in 15 of 26 patients (57.7%) in the VM-26 arm versus 23 of 27 (85.2%) for PFS, and in 14 (53.8%) versus 21 (77.8%) for OS.

### 3.3. Cox Regression and Sensitivity Analyses 

The treatment effect was preserved, and indeed strengthened, after adjustment (Table 3 and Table 4). On univariable analysis, the hazard ratio (HR) was 0.50 (95% CI 0.25–0.97, *p* = 0.041) for PFS (Table 3) and 0.47 (95% CI 0.23–0.98, *p* = 0.045) for OS (Table 4). In the pre-specified multivariable model—treatment plus three baseline covariates (KPS ≥ 90, glioblastoma IDH-wildtype, and recurrence surgery completed before chemotherapy)—the estimates moved further from unity, to 0.42 (95% CI 0.19–0.95, *p* = 0.037) for PFS (Table 3) and 0.32 (95% CI 0.12–0.85, *p* = 0.022) for OS (Table 4), with concordance indices of 0.588 and 0.598, respectively. None of the three covariates was independently associated with either endpoint (*p* = 0.813, 0.361 and 0.866 in the PFS model; *p* = 0.325, 0.195 and 0.876 in the OS model), indicating that the strengthening of the treatment estimate reflected the removal of baseline imbalance that had been masking, rather than creating, the apparent benefit. Of the remaining candidate variables, only male sex reached significance in univariable analysis for PFS (HR 0.49, 95% CI 0.25–0.95, *p* = 0.033; Table 3), and it was not significant for OS (HR 0.62, 95% CI 0.31–1.26, *p* = 0.188; Table 4); multiple lesions at recurrence showed a borderline association with shorter OS (HR 1.97, 95% CI 0.91–4.25, *p* = 0.084; Table 4). Because this variable was more frequent in the triplet group and could therefore confound the comparison, the primary multivariable model was refitted with multiple lesions at recurrence added as a fourth covariate: the treatment effect was preserved, and for overall survival it was strengthened (PFS HR 0.42, 95% CI 0.18–0.99, *p* = 0.048; OS HR 0.26, 95% CI 0.09–0.70, *p* = 0.008), while multiple lesions at recurrence emerged as an independent adverse prognostic factor for overall survival (HR 2.50, 95% CI 1.12–5.58, *p* = 0.025; Table S1).

Eleven pre-specified analysis strategies were used to probe the robustness of the treatment effect (Table S1). Every point estimate lay below 1—PFS HR 0.25–0.71 and OS HR 0.24–0.52—so no strategy reversed the direction of effect. The result was significant by univariable and multivariable Cox regression, by Cox regression stratified on KPS, by the time-varying coefficient model, and by landmark analysis at both 60 and 90 days for both endpoints; within the complete-case analysis the OS benefit remained significant after adjustment for MGMT (HR 0.34, 95% CI 0.13–0.87, *p* = 0.025). The three propensity score-based approaches produced estimates of the same magnitude and direction but did not reach significance: the score as a single covariate (PFS HR 0.63, *p* = 0.291; OS HR 0.39, *p* = 0.069), inverse probability of treatment weighting (0.60, *p* = 0.155; 0.52, *p* = 0.110) and 1:1 matching with stratified Cox regression (0.71, *p* = 0.566; 0.33, *p* = 0.179). The propensity score model itself (Table S2) showed that assignment to the teniposide-containing regimen was strongly determined by glioblastoma IDH-wildtype status (coefficient +2.247, *p* = 0.009) and by male sex (+2.057, *p* = 0.017), with recurrence surgery before chemotherapy contributing at a borderline level (−1.253, *p* = 0.095)—a pattern consistent with confounding by indication. Because the cohort comprised only 53 patients with 38 PFS and 35 OS events, the absence of significance in the weighted and matched analyses is most plausibly attributable to limited power rather than to attenuation of the effect; the direction and magnitude of the estimates were preserved throughout.

The proportional hazards assumption was satisfied for the treatment effect in both models (*p* = 0.173 for PFS and *p* = 0.293 for OS) and for all remaining covariates, with the single exception of KPS ≥ 90 in the PFS model, where a borderline violation was detected (χ^2^ = 4.718, *p* = 0.030) (Table S3). This did not appear to be a substantive violation. The KPS binary groups were not associated with outcome on log-rank testing (*p* = 0.914 for PFS and *p* = 0.842 for OS), and the treatment estimate was essentially unchanged when the assumption was addressed in four different ways (Table S4): stratification by KPS (PFS HR 0.42, *p* = 0.041; OS HR 0.35, *p* = 0.039); a time-varying coefficient model with log(t) centered on its median (0.41, *p* = 0.028; 0.35, *p* = 0.026); and landmark analyses at 60 days (0.28, *p* = 0.006; 0.24, *p* = 0.007) and 90 days (0.25, *p* = 0.006; 0.29, *p* = 0.021).

Molecular testing was not performed in 14 of 53 patients (26.4%). Because this missingness is structural—it reflects whether testing was carried out rather than a random process—markers were not imputed and a complete-case analysis was performed instead (Table S5). Restricting the analysis to the 39 tested patients reduced the number of events from 38 to 28 for PFS and from 35 to 25 for OS, and lowered the events-per-variable ratio from 9.5 and 8.8 to 7.0 and 6.3, respectively. The PFS estimate remained of similar magnitude but lost significance (HR 0.51, 95% CI 0.20–1.29, *p* = 0.155), whereas the OS estimate stayed of similar magnitude (HR 0.32, *p* = 0.055) and became significant after additional adjustment for MGMT promoter methylation (HR 0.34, 95% CI 0.13–0.87, *p* = 0.025). In the 14 untested patients the treatment effect could not be estimated meaningfully (OS HR 5.77, 95% CI 0.33–102.5), a confidence interval spanning three orders of magnitude. Tested and untested patients differed on several baseline characteristics (Table S6): those who underwent testing were more likely to have had gross total resection at initial surgery (SMD +0.626) and to receive the teniposide-containing regimen (+0.587), and less likely to have had recurrence surgery before chemotherapy (−0.582); they were also younger (−0.501) and less often male (−0.424). This pattern indicates selection towards a more favorable-risk population, yet testing status itself was not associated with survival (median PFS 158 vs. 174 days; median OS 247 vs. 227 days; log-rank *p* = 0.266). Finally, moderate collinearity was present among the molecular markers (Table S7; MGMT versus CDKN2A/B r = +0.858 with VIF 6.21 and 5.45; MGMT versus TERT r = +0.825; TERT versus CDKN2A/B r = +0.792; glioblastoma IDH-wildtype versus MGMT r = −0.721), which is why the multivariable models used the integrated diagnosis rather than individual molecular markers.

Because the inclusion of IDH-mutant tumors may be questioned under the WHO CNS5 classification, the survival analyses were repeated in the molecularly defined subset of patients with glioblastoma, IDH-wildtype, WHO grade 4 (*n* = 33; 20 treated with the triplet and 13 with the pooled control); the 6 patients with astrocytoma, IDH-mutant, WHO grade 4 and the 14 patients without molecular testing were excluded (Table S10). The estimates in this subset were in the same direction as in the full cohort: median PFS was 7.8 months (95% CI 3.9–not reached) versus 4.8 months (3.8–6.7) (log-rank *p* = 0.021; unadjusted HR 0.35, 95% CI 0.14–0.89; Cox *p* = 0.027), and median OS was 12.7 months (8.4–15.4) versus 7.8 months (6.8–10.0) (log-rank *p* = 0.012; unadjusted HR 0.32, 95% CI 0.12–0.82; Cox *p* = 0.017). Restricting the analysis further to the concurrently treated IDH-wildtype patients (20 vs. 9) preserved the direction of the effect (PFS HR 0.42, 95% CI 0.15–1.17; OS HR 0.39, 95% CI 0.13–1.13), without reaching significance. The IDH-mutant cases and the untested cases therefore do not appear to account for the observed difference; the association was not attenuated when they were excluded.

### 3.4. Analyses Accounting for Treatment Era

Because teniposide became available again in China only after 2022, all patients treated with the triplet were treated during 2023–2026, whereas the comparator cohort spans both periods. Restricting the analysis to the two concurrently treated overlap-period cohorts isolates the effect of adding teniposide from that of treatment era (Table S8). Median PFS was 7.8 months (95% CI 4.9–10.9) with the triplet versus 6.3 months (95% CI 2.9–8.3) with concurrent TMZ plus bevacizumab (HR 0.62, 95% CI 0.28–1.36; log-rank *p* = 0.228), and median OS was 12.7 months (95% CI 8.4–15.4) versus 9.7 months (95% CI 5.5–10.3) (HR 0.60, 95% CI 0.25–1.45; log-rank *p* = 0.252); the 12-month OS rate was 50.7% versus 12.8%. After additional adjustment for multiple lesions at recurrence, the estimates were unchanged in direction (PFS HR 0.57, 95% CI 0.22–1.46; OS HR 0.46, 95% CI 0.15–1.39). Only the number of radiotherapy fractions and the number of prior adjuvant TMZ cycles differed significantly between these two concurrent groups (*p* = 0.020 and *p* = 0.025, respectively); all remaining baseline comparisons had *p* ≥ 0.082.

To quantify the era effect itself, the same TMZ plus bevacizumab regimen was compared across the two periods. Outcome was numerically better in the later period for both endpoints (PFS median 6.3 vs. 4.7 months, HR 0.76, 95% CI 0.33–1.73, *p* = 0.507; OS median 9.7 vs. 8.2 months, HR 0.82, 95% CI 0.34–1.95, *p* = 0.648), and the two control eras were balanced apart from the extent of resection at recurrence (partial resection, *p* = 0.028; all remaining comparisons had *p* ≥ 0.057). Because this secular trend favors the later era, from which the entire triplet group was drawn, any residual era effect would tend to inflate rather than diminish the pooled estimate. The pooled estimates should therefore be read as an upper bound and the era-matched estimates as the more conservative ones; the loss of statistical significance in the latter reflects the size of the concurrent control group (*n* = 15) rather than a reversal in the direction of the effect.

### 3.5. Tumor Response

To separate the effect of adding teniposide from that of treatment era, efficacy endpoints were compared across three groups: patients treated with TMZ + Bev during the non-overlap period (2020–2022; *n* = 12), patients treated with TMZ + Bev during the overlap period (2023–2026; *n* = 15), and patients treated with VM26 + TMZ + Bev during the overlap period (*n* = 26), all analyzed on an intention-to-treat basis with progression or death before the assessment window counted as PD (Table 5). Within the overlap period, where both regimens were available and patients were treated concurrently, the objective response rate was 26.9% (7/26) in the VM26 group versus 13.3% (2/15) in the TMZ + Bev group, a difference of +13.6 percentage points (two-sided *p* = 0.445); the median (IQR) interval from the index date to response assessment was comparable between the two groups (4.90 [2.88–5.89] vs. 5.22 [3.33–6.93] months; difference −0.33 months, *p* = 0.507), indicating that the apparent response difference was not attributable to unequal assessment windows. Corticosteroid therapy during salvage treatment was recorded for every patient and was given with similar frequency in the two concurrently treated groups (76.9% [20/26] vs. 66.7% [10/15]; *p* = 0.491) and against the pooled control group (76.9% vs. 66.7%; *p* = 0.544), so it did not differ between the arms at the time of response assessment. Dexamethasone was used in 34 patients (10 mg daily for 14 days in 18 and 5 mg daily for 7 days in 16) and methylprednisolone in 4 (40 mg daily for 5 days). Corticosteroid use was not associated with PFS (HR 1.13, 95% CI 0.55–2.34; *p* = 0.738) or with overall survival (HR 1.27, 95% CI 0.57–2.80; *p* = 0.559). The disease control rate was 80.8% (21/26) with VM26 versus 86.7% (13/15) with TMZ + Bev (−5.9 percentage points, *p* = 1.000), whereas early progression within 2 months occurred in 15.4% (4/26) versus 6.7% (1/15) of patients (+8.7 percentage points, *p* = 0.636). No efficacy endpoint differed significantly between the two overlap-period groups (all *p* ≥ 0.218). When the same regimen was compared across periods, TMZ + Bev achieved a numerically higher disease control rate during the overlap period than during the non-overlap period (86.7% vs. 66.7%; +20.0 percentage points, *p* = 0.357), indicating a secular improvement in disease control over time; because this trend favors the later-treated regimen, comparing the VM26 regimen with the historical control would have inflated any apparent benefit, and the within-era comparison reported here is therefore the more conservative estimate. Finally, because the primary efficacy analysis compared the VM26 regimen with all TMZ + Bev patients combined, the two TMZ + Bev groups were also pooled into a single control group spanning both periods (2020–2026; *n* = 27) and compared with the overlap-period VM26 regimen, again on an intention-to-treat basis (Table 5). In this pooled control comparison, the objective response rate was 26.9% (7/26) with VM26 versus 14.8% (4/27) with TMZ + Bev (+12.1 percentage points, *p* = 0.327), the disease control rate was 80.8% (21/26) versus 77.8% (21/27) (+3.0 percentage points, *p* = 1.000), early progression within 2 months occurred in 15.4% (4/26) versus 3.7% (1/27) of patients (+11.7 percentage points, *p* = 0.192), and the median (IQR) interval from the index date to response assessment was 4.90 (2.88–5.89) versus 5.13 (4.06–6.87) months (difference −0.23 months, *p* = 0.328). None of these differences was statistically significant. Overall, these exploratory analyses did not demonstrate a statistically significant efficacy advantage for the VM26-containing regimen, and the study was not powered to detect moderate differences in the magnitude that would be clinically relevant.

### 3.6. Treatments After Progression

Of the 53 patients, 15 (28.3%) had not progressed at the data cut-off and therefore received no post-progression treatment. Among the 38 patients who progressed, subsequent management consisted of continuation of the same bevacizumab-based regimen in 14, a further line of systemic therapy in 9, best supportive care in 9 and local therapy (repeat resection or re-irradiation) in 6. Expressed as a proportion of the patients who progressed in each group, the distribution was similar between the triplet group and the concurrently treated control cohort (continuation of the same regimen 46.7% vs. 27.3%; systemic therapy 33.3% vs. 9.1%; best supportive care 13.3% vs. 54.5%; local therapy 6.7% vs. 9.1%; Monte-Carlo exact *p* = 0.165) and between the triplet group and the pooled control group (46.7% vs. 30.4%; 33.3% vs. 17.4%; 13.3% vs. 30.4%; 6.7% vs. 21.7%; Monte-Carlo exact *p* = 0.308). Because treatment after progression was recorded for every patient and did not differ significantly between the groups, subsequent therapy is unlikely to account for the observed difference in survival.

### 3.7. Safety

The safety analysis included all 53 patients, with adverse events graded according to CTCAE version 5.0 and summarized as the worst grade recorded for each patient during salvage therapy (Table 6). Across the whole cohort, 19 of 53 patients (35.8%) experienced at least one adverse event of any grade and 5 (9.4%) experienced an event of grade ≥ 3; no grade 5 event was recorded. Within the overlap period, in which both regimens were available and patients were treated concurrently, any-grade adverse events occurred in 46.2% (12/26) of patients receiving VM26 + TMZ + Bev versus 26.7% (4/15) of those receiving TMZ + Bev (+19.5 percentage points, *p* = 0.322), and grade ≥ 3 events in 11.5% (3/26) versus 6.7% (1/15) (+4.9 percentage points, *p* = 1.000). The numerically higher overall toxicity in the VM26 group was driven predominantly by hematologic events (42.3% vs. 26.7%; +15.6 percentage points, *p* = 0.502), and specifically by leukopenia and neutropenia, each of which occurred in 26.9% (7/26) of patients in the VM26 group compared with none of the 15 patients in the overlap-period TMZ + Bev group (+26.9 percentage points; two-sided *p* = 0.035 for each). However, after correction for multiplicity across the 10 individual adverse events using the Benjamini–Hochberg procedure, neither difference remained statistically significant (Benjamini–Hochberg-adjusted q = 0.177 for both). When the events were analyzed by grade, the excess in the triplet group was predominantly of low grade: grade 1–2 leukopenia occurred in 19.2% (5/26) of patients versus none of the 15 concurrently treated controls (*p* = 0.139) and grade 1–2 neutropenia in 15.4% (4/26) versus none (*p* = 0.278), whereas grade ≥ 3 events were uncommon and did not differ significantly (leukopenia, 7.7% [2/26] vs. 0%, *p* = 0.524; neutropenia, 11.5% [3/26] vs. 0%, *p* = 0.287). All grade ≥ 3 events in the cohort were hematologic—leukopenia (grade 3, 3 patients), neutropenia (grade 3, 3 patients) and lymphopenia (grade 3 in one patient and grade 4 in one patient, the only grade 4 event, in the triplet arm)—no grade 5 event occurred, and every non-hematologic event was of grade 1 or 2. Non-hematologic toxicity was uncommon and did not differ meaningfully between the two groups (23.1% vs. 6.7%; +16.4 percentage points, *p* = 0.232), and the highest CTCAE grade recorded per patient was low and comparable (median 0 [IQR 0.0–1.0] vs. 0 [0.0–0.5]; *p* = 0.300). 

Consistent with this, toxicity-driven treatment modification was infrequent: dose reduction was required in 11.5% (3/26) versus 6.7% (1/15) of patients (+4.9 percentage points, *p* = 1.000), and treatment discontinuation because of an adverse event in 11.5% (3/26) versus 6.7% (1/15) (+4.9 percentage points, *p* = 1.000). Salvage chemotherapy was given in 28-day cycles in all 53 patients, and the reason for stopping treatment was recorded for every patient. Within the overlap period, treatment was discontinued because of progression in 46.2% (12/26) of patients receiving the VM26 triplet versus 66.7% (10/15) of those receiving TMZ + Bev (*p* = 0.330) and because of an adverse event in 11.5% (3/26) versus 6.7% (1/15) (*p* = 1.000), whereas 42.3% (11/26) versus 26.7% (4/15) were still receiving treatment at the data cut-off (*p* = 0.502). Across the whole cohort, treatment was discontinued because of progression in 34 patients (64.2%), because of an adverse event in 4 (7.5%), and 15 patients (28.3%) were still receiving treatment. 

Importantly, comparing the same TMZ + Bev regimen across the two treatment periods revealed no evidence of a shift in toxicity over time (any-grade adverse event +1.7 percentage points, *p* = 1.000; grade ≥ 3 adverse event −1.7 percentage points, *p* = 1.000; leukopenia and neutropenia −8.3 percentage points, *p* = 0.444), indicating that the differences observed between the two overlap-period groups were unlikely to be explained by treatment era. In a secondary analysis that combined the two TMZ + Bev control cohorts irrespective of treatment period (*n* = 27) and therefore does not adjust for era, the pattern of results was unchanged: any-grade adverse events remained numerically more frequent with VM26 + TMZ + Bev (46.2% vs. 25.9%; +20.2 percentage points, *p* = 0.158), and leukopenia and neutropenia were again the only events with a nominally significant difference (26.9% vs. 3.7% for each; +23.2 percentage points, *p* = 0.024), which did not persist after Benjamini–Hochberg correction (Benjamini–Hochberg-adjusted q = 0.122); grade ≥ 3 events (+4.1 percentage points, *p* = 0.669), dose reduction (+4.1 percentage points, *p* = 0.669) and treatment discontinuation because of an adverse event (+7.8 percentage points, *p* = 0.351) likewise remained numerically higher in the VM26 group without reaching statistical significance. Taken together, adding teniposide to TMZ + Bev was associated with a higher incidence of hematologic toxicity, chiefly leukopenia and neutropenia. This difference did not remain statistically significant after correction for multiplicity, but it was consistent in direction in both the era-matched and the pooled comparisons, it was predominantly of low grade, and myelosuppression is the toxicity expected of a topoisomerase II inhibitor. The toxicity profile of the triplet should therefore not be read as showing no meaningful increase in toxicity; blood counts should be monitored before each cycle, with dose modification as required (Table 6).

## 4. Discussion

Despite current standard-of-care therapies, the prognosis for GBM patients remains dismal, driving intensive efforts to integrate novel therapeutic agents into clinical regimens. After VM-26 was reintroduced in China in 2022, we implemented VM-26-based therapy for recurrent grade 4 glioma.

In this single-center, real-world exploratory study of patients with recurrent grade 4 glioma, the addition of teniposide to a backbone of TMZ plus Bev was associated with longer PFS and OS and a numerically higher ORR, without a significant increase in grade ≥3 toxicity, although any-grade leukopenia and neutropenia were more frequent and should be anticipated. Specifically, median PFS was 7.8 months versus 5.1 months (log-rank *p* = 0.037; unadjusted HR 0.50, 95% CI, 0.25–0.97), median OS was 12.7 months versus 8.5 months (log-rank *p* = 0.042; unadjusted HR 0.47, 95% CI, 0.23–0.98), and the ORR was 26.9% versus 14.8% (*p* = 0.327) in the VM26 + TMZ + Bev and TMZ + Bev arms, respectively. Multivariable adjustment strengthened these estimates, whereas the propensity score-based analyses yielded estimates of the same direction that did not reach significance, consistent with the limited number of events. These findings suggest that reintroducing teniposide may add meaningful clinical benefit to Bev-based salvage therapy for recurrent grade 4 glioma.

Recurrent GBM remains a therapeutic challenge. After first-line treatment with maximal safe resection followed by radiotherapy with concomitant and adjuvant temozolomide [17], salvage options are limited and generally offer only modest activity. Bevacizumab, a humanized monoclonal antibody against vascular endothelial growth factor, received accelerated approval for recurrent GBM on the basis of high radiographic response rates and prolonged PFS in uncontrolled phase II trials [18]. In the BRAIN study, bevacizumab alone and in combination with irinotecan produced objective response rates of 28.2% and 37.8%, with median OS of 9.2 and 8.7 months, respectively [18]. However, whether bevacizumab confers an OS benefit remains controversial; the phase III EORTC 26101 trial found that adding bevacizumab to lomustine prolonged PFS (4.2 vs. 1.5 months) but did not improve OS (9.1 vs. 8.6 months; HR 0.95) [19].

Teniposide (VM-26) is a podophyllotoxin-derived topoisomerase II inhibitor. Its activity in malignant glioma has been recognized for decades, primarily in combination with nitrosoureas. In the NOA-01 trial, first-line nimustine plus teniposide yielded a median OS of 17.3 months in the glioblastoma subgroup [20]. In the recurrent setting, the retrospective experience of nimustine plus teniposide reported a median OS of 6 months and a 6-month PFS of 29%, with an acceptable toxicity profile apart from myelosuppression [21]. The present study adds to this evidence by showing that teniposide can be combined with the contemporary bevacizumab-based backbone, with an objective response rate of 26.9% that is comparable to that reported for previous teniposide- or bevacizumab-containing regimens in recurrent GBM. Notably, teniposide was reintroduced into production in 2022, renewing interest in this agent as a repurposed component of combination therapy.

Beyond bevacizumab and lomustine, several other salvage options have been evaluated in recurrent GBM. Regorafenib, an oral multikinase inhibitor of angiogenic, stromal and oncogenic receptor tyrosine kinases, was compared with lomustine in the randomized phase 2 REGOMA trial, in which it prolonged median overall survival (7.4 vs. 5.6 months; HR 0.50, 95% CI 0.33–0.75; *p* = 0.0009) at the cost of more frequent grade 3–4 treatment-related adverse events (56% vs. 40%) [22]. Regorafenib has since been incorporated into several European treatment pathways, whereas repeat resection and re-irradiation remain alternatives in selected patients; nevertheless, no single standard of care has been established for recurrence [23,24].

In the absence of a clearly superior salvage regimen, the present results are best interpreted against the range of outcomes reported for other second-line options. The median overall survival of 12.7 months observed with the teniposide triplet compares favorably with that reported for bevacizumab alone (9.2 months) and bevacizumab plus irinotecan (8.7 months) [18], lomustine plus bevacizumab (9.1 months) [19], regorafenib (7.4 months) [22] and lomustine alone (5.6–8.6 months) [19,22]. A similar pattern was seen for response, the objective response rate of 26.9% lying close to the lower end of the range reported for bevacizumab-containing regimens (28.2–37.8%) [18]. In the BELOB trial, the combination of bevacizumab and lomustine achieved a 9-month overall survival rate of 59%, which was regarded as sufficiently promising to justify a phase 3 trial [25]. These cross-study comparisons must nevertheless be interpreted with caution, because our cohort was small and single-center, part of the comparator group was treated in an earlier era, eligibility criteria and baseline prognostic factors differed from those of the trials cited, and the confidence intervals of the propensity score-based estimates still included unity. They support the plausibility of a clinically meaningful contribution from teniposide rather than superiority over any specific alternative.

The hematologic toxicity of this regimen should be anticipated rather than described only as an absence of excess high-grade toxicity. Any-grade adverse events were more frequent in the teniposide group, and leukopenia and neutropenia were each recorded in 26.9% (7/26) of patients receiving the triplet compared with none of the 15 concurrently treated controls, a difference that could not be attributed to treatment era. Neither difference remained significant after correction for multiplicity (Benjamini–Hochberg-adjusted q = 0.177); however, multiplicity correction limits the risk of false-positive findings and does not imply that the signal is absent. The direction of the effect was consistent in both the era-matched and the pooled comparisons, and myelosuppression is the expected toxicity of a topoisomerase II inhibitor. Clinicians using this regimen should therefore anticipate leukopenia and neutropenia, monitor blood counts before each cycle, and apply dose modification as required. In this cohort the signal was manageable, dose reduction and treatment discontinuation each being required in 11.5% of patients receiving the triplet, but it should be weighed against the survival signal when this regimen is considered for an individual patient.

Several limitations warrant caution. First, this was a single-center, retrospective, exploratory analysis with a modest sample size (*n* = 53), and the absence of randomization precludes causal inference. In addition, five patients who started salvage chemotherapy but discontinued it before completing two cycles, without documented progression or death, were not included, because the cohort was defined by a minimum of two cycles. The direction of the resulting bias cannot be determined with certainty, but because early treatment-related toxicity was more frequent with the triplet, this criterion may have removed early treatment-related events preferentially from the triplet arm; the survival comparison should therefore be regarded as potentially optimistic. Second, molecular data were unavailable for a substantial proportion of patients, and this is a limitation distinct from sample size. Testing was not performed in 15.4% of the triplet group (4 of 26 patients) and 37.0% of the pooled control group (10 of 27) because archival tissue was unavailable or testing had not been requested, and the proportion untested was uneven across the treatment eras (58.3% of the non-overlap-period control cohort versus 20.0% of the overlap-period control cohort). The comparison of IDH, TERT, MGMT, CDKN2A/B and 1p/19q status therefore rests on the patients who were tested only, and no imputation was performed (Section 2.8.6). Two observations limit the impact of this on the treatment comparison: within the era-matched comparison the two concurrently treated groups were tested at a similar rate (84.6% vs. 80.0%; *p* = 0.693), and the complete-case analysis restricted to the 39 tested patients preserved the direction of the treatment effect (Table S5), although the number of events fell from 38 to 28 for PFS and from 35 to 25 for OS and the confidence intervals widened accordingly. Third, and most importantly, because teniposide production resumed only in 2022, all patients treated with the triplet were treated during 2023–2026, whereas the comparator cohort spans both periods; treatment era therefore remains the principal threat to the internal validity of these findings. When the analysis was restricted to the two concurrently treated cohorts, the direction of the treatment effect was preserved for both endpoints (PFS HR 0.62, 95% CI 0.28–1.36; OS HR 0.60, 95% CI 0.25–1.45), but neither estimate reached significance and both confidence intervals included unity. The cross-era comparison of the identical control regimen indicated that outcome was, if anything, better in the later period (PFS HR 0.76; OS HR 0.82), so that this source of bias would tend to inflate rather than diminish the pooled estimate. The pooled estimate should therefore be regarded as an upper bound and the era-matched estimate as the conservative one. Fourth, radiographic responses were assessed locally rather than by blinded central review, and because bevacizumab reduces contrast enhancement by normalizing tumor vasculature, pseudoresponse cannot be excluded in a retrospective single-center series of this kind. Three considerations limit the extent to which this could account for the present findings. Bevacizumab was administered in both arms, so any pseudoresponse attributable to the antiangiogenic agent would affect the two groups similarly and would tend to dilute rather than create a between-arm difference. The difference in overall survival, which is not derived from imaging, was of similar magnitude to that in progression-free survival and cannot be explained by an imaging phenomenon. And the objective response rate did not differ significantly between the groups (26.9% vs. 13.3%; *p* = 0.445), so no conclusion in this report rests on the response rate. Fifth, residual confounding by indication cannot be excluded. Patients receiving the triplet had received fewer adjuvant TMZ cycles (median 1.0 vs. 5.5; *p* = 0.003) and less often underwent re-resection at recurrence (23.1% vs. 51.9%; *p* = 0.031), whereas multiple lesions at recurrence were numerically more frequent in the triplet arm without reaching significance. This matters because re-operation at recurrence is itself an established favorable prognostic factor. Two considerations limit its impact on the treatment comparison. Re-resection at recurrence before the start of salvage chemotherapy was one of the three covariates selected a priori for the primary multivariable model, and both re-resection at recurrence and extent of resection at initial surgery were included in the propensity score model. In addition, within the era-matched comparison the two concurrently treated cohorts did not differ significantly in re-resection rate (40.0% vs. 23.1%; *p* = 0.300), so the imbalance is in large part a feature of the historical control cohort rather than of the treatment decision itself. Sixth, adverse events were extracted retrospectively from medical records and the frequency of laboratory monitoring was not standardized, so toxicity may be underestimated. Seventh, the survival analyses were based on a single pre-specified comparison and were not adjusted for multiple comparisons, whereas the ten individual adverse events were corrected using the Benjamini–Hochberg procedure, after which leukopenia and neutropenia no longer remained significant (q = 0.177). These factors may inflate the observed treatment effects and limit generalizability.

The statistical fragility of these results warrants explicit comment. With 38 progression-free and 35 overall survival events distributed across 53 patients, both primary comparisons rest on a small number of events, and the upper limits of the hazard ratio confidence intervals approached unity (0.97 for PFS and 0.98 for OS). The results are fragile in the sense that they would not withstand reclassification of individual patient outcomes: altering the recorded progression or death of a very small number of patients would render either difference non-significant. We therefore do not present these findings as an established survival benefit, and causal language has been avoided throughout. What supports our interpretation is the consistency rather than the precision of the evidence. All eleven pre-specified analyses pointed in the same direction, with hazard ratios between 0.24 and 0.71; adjustment for baseline covariates moved the estimates further from unity rather than towards it; the proportional hazards assumption was satisfied for the treatment effect; and landmark analyses restricted to patients who survived beyond 60 or 90 days produced the smallest *p* values in the series (0.006 to 0.021). These observations describe an internally consistent signal whose precision is limited by the size of the cohort, and a prospective randomized study remains necessary to determine whether the difference is real.

In conclusion, the addition of teniposide to TMZ plus Bev was associated with longer PFS and OS and a numerically higher ORR in patients with recurrent grade 4 glioma, without a significant increase in grade ≥ 3 toxicity, although any-grade leukopenia and neutropenia were more frequent and should be anticipated; treatment era could not be fully separated from treatment effect, and the propensity score analyses did not reach significance. Because treatment was not assigned at random, these associations cannot be taken to establish that teniposide caused the observed differences. Although these real-world, exploratory findings are hypothesis-generating, they provide a rationale for prospective, randomized evaluation of teniposide-based combinations as salvage therapy for recurrent grade 4 glioma.

## Figures and Tables

**Table 1:** Baseline characteristics of the study population.

Characteristic	Non-Overlap Period (2020–2022) TMZ + Bev (*n* = 12)	Overlap Period (2023–2026) TMZ + Bev (*n* = 15)	Pooled Control (2020–2026) TMZ + Bev (*n* = 27)	Overlap Period (2023–2026) VM26 + TMZ + Bev (*n* = 26)	Between-Group Difference a (*p* Value)	Across-Period Change b (*p* Value)	VM26 + TMZ + Bev vs. Pooled Control c (*p* Value)
**Patient and tumor characteristics at baseline**							
Age, years	56.5 (50.2–63.5)	62.0 (49.0–70.0)	62.0 (49.5–69.0)	51.5 (41.2–67.8)	−10.5/(*p* = 0.440)	+5.5/(*p* = 0.449)	−10.5/(*p* = 0.433)
**Sex**							
Male	5 (41.7)	5 (33.3)	10 (37.0)	16 (61.5)	+28.2/(*p* = 0.082)	−8.4/(*p* = 0.706)	+24.5/(*p* = 0.074)
Female	7 (58.3)	10 (66.7)	17 (63.0)	10 (38.5)	−28.2/(*p* = 0.082)	+8.4/(*p* = 0.706)	−24.5/(*p* = 0.074)
Body weight, kg	60.9 ± 13.9	63.3 ± 13.2	62.3 ± 13.3	67.1 ± 12.7	+3.8/(*p* = 0.370)	+2.4/(*p* = 0.648)	+4.8/(*p* = 0.180)
Height, m	1.62 ± 0.08	1.64 ± 0.07	1.63 ± 0.07	1.68 ± 0.08	+0.04/(*p* = 0.120)	+0.02/(*p* = 0.532)	+0.05/(*p* = 0.030)
Body mass index, kg/m^2^	22.9 ± 3.7	23.4 ± 3.9	23.2 ± 3.8	23.8 ± 4.0	+0.4/(*p* = 0.810)	+0.5/(*p* = 0.745)	+0.6/(*p* = 0.623)
**Comorbidities**							
Any comorbidity	7 (58.3)	6 (40.0)	13 (48.1)	12 (46.2)	+6.2/(*p* = 0.702)	−18.3/(*p* = 0.343)	−1.9/(*p* = 0.884)
Hypertension	5 (41.7)	5 (33.3)	10 (37.0)	10 (38.5)	+5.2/(*p* = 0.743)	−8.4/(*p* = 0.706)	+1.5/(*p* = 0.915)
Diabetes mellitus	2 (16.7)	3 (20.0)	5 (18.5)	3 (11.5)	−8.5/(*p* = 0.651)	+3.3/(*p* = 1.000)	−7.0/(*p* = 0.704)
Hyperlipidemia	0 (0.0)	3 (20.0)	3 (11.1)	1 (3.8)	−16.2/(*p* = 0.130)	+20.0/(*p* = 0.231)	−7.3/(*p* = 0.610)
Prior cerebral infarction	0 (0.0)	3 (20.0)	3 (11.1)	2 (7.7)	−12.3/(*p* = 0.336)	+20.0/(*p* = 0.231)	−3.4/(*p* = 1.000)
**Initial treatment and disease history**							
**Initial treatment regimen**							
Surgery + RT + concurrent TMZ	7 (58.3)	11 (73.3)	18 (66.7)	23 (88.5)	+15.2/(*p* = 0.390)	+15.0/(*p* = 0.448)	+21.8/(*p* = 0.058)
Surgery + TMZ	5 (41.7)	3 (20.0)	8 (29.6)	3 (11.5)	−8.5/(*p* = 0.651)	−21.7/(*p* = 0.398)	−18.1/(*p* = 0.104)
Surgery + RT	0 (0.0)	1 (6.7)	1 (3.7)	0 (0.0)	−6.7/(*p* = 0.366)	+6.7/(*p* = 1.000)	−3.7/(*p* = 1.000)
**Extent of initial resection**							
Gross total resection	2 (16.7)	7 (46.7)	9 (33.3)	9 (34.6)	−12.1/(*p* = 0.446)	+30.0/(*p* = 0.217)	+1.3/(*p* = 0.922)
Subtotal resection	7 (58.3)	5 (33.3)	12 (44.4)	11 (42.3)	+9.0/(*p* = 0.570)	−25.0/(*p* = 0.194)	−2.1/(*p* = 0.875)
Partial resection	3 (25.0)	2 (13.3)	5 (18.5)	4 (15.4)	+2.1/(*p* = 1.000)	−11.7/(*p* = 0.628)	−3.1/(*p* = 1.000)
Biopsy	0 (0.0)	1 (6.7)	1 (3.7)	2 (7.7)	+1.0/(*p* = 1.000)	+6.7/(*p* = 1.000)	+4.0/(*p* = 0.610)
Received radiotherapy as part of initial treatment	7 (58.3)	12 (80.0)	19 (70.4)	23 (88.5)	+8.5/(*p* = 0.651)	+21.7/(*p* = 0.398)	+18.1/(*p* = 0.104)
Radiotherapy fractions, n d	30 (25–30)	29 (21–30)	30 (21–30)	30 (30–30)	+1.0/(*p* = 0.020)	−1.0/(*p* = 0.653)	0.0/(*p* = 0.031)
Adjuvant TMZ cycles, n	5.5 (1.0–6.0)	5.0 (1.0–6.0)	5.5 (1.0–6.0)	1.0 (1.0–2.5)	−4.0/(*p* = 0.025)	−0.5/(*p* = 0.623)	−4.5/(*p* = 0.003)
Time from initial treatment to recurrence, months	9.2 (8.1–26.4)	9.4 (5.4–17.1)	9.4 (7.3–21.7)	10.3 (5.9–17.8)	+0.9/(*p* = 1.000)	+0.2/(*p* = 0.479)	+0.9/(*p* = 0.471)
**Characteristics at recurrence**							
**Number of recurrences**							
1	8 (66.7)	13 (86.7)	21 (77.8)	20 (76.9)	−9.8/(*p* = 0.687)	+20.0/(*p* = 0.357)	−0.9/(*p* = 0.941)
2	4 (33.3)	2 (13.3)	6 (22.2)	6 (23.1)	+9.8/(*p* = 0.687)	−20.0/(*p* = 0.357)	+0.9/(*p* = 0.941)
Multiple recurrent tumors	2 (16.7)	4 (26.7)	6 (22.2)	10 (38.5)	+11.8/(*p* = 0.443)	+10.0/(*p* = 0.662)	+16.3/(*p* = 0.198)
**Tumor location at recurrence**							
Left hemisphere	6 (50.0)	7 (46.7)	13 (48.1)	8 (30.8)	−15.9/(*p* = 0.309)	−3.3/(*p* = 0.863)	−17.3/(*p* = 0.196)
Right hemisphere	5 (41.7)	5 (33.3)	10 (37.0)	12 (46.2)	+12.9/(*p* = 0.422)	−8.4/(*p* = 0.706)	+9.2/(*p* = 0.501)
Other or midline	1 (8.3)	3 (20.0)	4 (14.8)	6 (23.1)	+3.1/(*p* = 1.000)	+11.7/(*p* = 0.605)	+8.3/(*p* = 0.501)
Surgery at recurrence	8 (66.7)	6 (40.0)	14 (51.9)	6 (23.1)	−16.9/(*p* = 0.300)	−26.7/(*p* = 0.168)	−28.8/(*p* = 0.031)
**Extent of resection at recurrence**							
No surgery	4 (33.3)	9 (60.0)	13 (48.1)	20 (76.9)	+16.9/(*p* = 0.300)	+26.7/(*p* = 0.168)	+28.8/(*p* = 0.031)
Gross total resection	2 (16.7)	2 (13.3)	4 (14.8)	4 (15.4)	+2.1/(*p* = 1.000)	−3.4/(*p* = 1.000)	+0.6/(*p* = 1.000)
Subtotal resection	2 (16.7)	3 (20.0)	5 (18.5)	1 (3.8)	−16.2/(*p* = 0.130)	+3.3/(*p* = 1.000)	−14.7/(*p* = 0.192)
Partial resection	4 (33.3)	0 (0.0)	4 (14.8)	1 (3.8)	+3.8/(*p* = 1.000)	−33.3/(*p* = 0.028)	−11.0/(*p* = 0.351)
Biopsy	0 (0.0)	1 (6.7)	1 (3.7)	0 (0.0)	−6.7/(*p* = 0.366)	+6.7/(*p* = 1.000)	−3.7/(*p* = 1.000)
Re-resection completed before study chemotherapy	6 (50.0)	5 (33.3)	11 (40.7)	5 (19.2)	−14.1/(*p* = 0.453)	−16.7/(*p* = 0.452)	−21.5/(*p* = 0.088)
Cycles of salvage chemotherapy, *n*	4.5 (4.0–6.0)	5.0 (3.0–6.0)	5.0 (4.0–6.0)	5.5 (3.2–6.0)	+0.5/(*p* = 0.826)	+0.5/(*p* = 0.984)	+0.5/(*p* = 0.800)
**Molecular pathology**							
Molecular testing performed e	5 (41.7)	12 (80.0)	17 (63.0)	22 (84.6)	+4.6/(*p* = 0.693)	+38.3/(*p* = 0.057)	+21.6/(*p* = 0.074)
**IDH status**							
Mutant	1 (8.3)	3 (20.0)	4 (14.8)	2 (7.7)	−12.3/(*p* = 0.336)	+11.7/(*p* = 0.605)	−7.1/(*p* = 0.669)
Wildtype	4 (33.3)	9 (60.0)	13 (48.1)	20 (76.9)	+16.9/(*p* = 0.300)	+26.7/(*p* = 0.168)	+28.8/(*p* = 0.031)
Not tested	7 (58.3)	3 (20.0)	10 (37.0)	4 (15.4)	−4.6/(*p* = 0.693)	−38.3/(*p* = 0.057)	−21.6/(*p* = 0.074)
**MGMT promoter methylation**							
Methylated	1 (8.3)	4 (26.7)	5 (18.5)	8 (30.8)	+4.1/(*p* = 1.000)	+18.4/(*p* = 0.342)	+12.3/(*p* = 0.300)
Unmethylated	4 (33.3)	8 (53.3)	12 (44.4)	14 (53.8)	+0.5/(*p* = 0.975)	+20.0/(*p* = 0.299)	+9.4/(*p* = 0.494)
Not tested	7 (58.3)	3 (20.0)	10 (37.0)	4 (15.4)	−4.6/(*p* = 0.693)	−38.3/(*p* = 0.057)	−21.6/(*p* = 0.074)
**TERT promoter mutation**							
Mutant	2 (16.7)	3 (20.0)	5 (18.5)	9 (34.6)	+14.6/(*p* = 0.480)	+3.3/(*p* = 1.000)	+16.1/(*p* = 0.184)
Wildtype	3 (25.0)	9 (60.0)	12 (44.4)	13 (50.0)	−10.0/(*p* = 0.536)	+35.0/(*p* = 0.069)	+5.6/(*p* = 0.685)
Not tested	7 (58.3)	3 (20.0)	10 (37.0)	4 (15.4)	−4.6/(*p* = 0.693)	−38.3/(*p* = 0.057)	−21.6/(*p* = 0.074)
**CDKN2A/B homozygous deletion**							
Present	1 (8.3)	3 (20.0)	4 (14.8)	6 (23.1)	+3.1/(*p* = 1.000)	+11.7/(*p* = 0.605)	+8.3/(*p* = 0.501)
Absent	4 (33.3)	9 (60.0)	13 (48.1)	16 (61.5)	+1.5/(*p* = 0.923)	+26.7/(*p* = 0.168)	+13.4/(*p* = 0.328)
Not tested	7 (58.3)	3 (20.0)	10 (37.0)	4 (15.4)	−4.6/(*p* = 0.693)	−38.3/(*p* = 0.057)	−21.6/(*p* = 0.074)
**1p/19q codeletion f**							
Non-codeleted	5 (41.7)	12 (80.0)	17 (63.0)	21 (80.8)	+0.8/(*p* = 1.000)	+38.3/(*p* = 0.057)	+17.8/(*p* = 0.150)
Not tested	7 (58.3)	3 (20.0)	10 (37.0)	5 (19.2)	−0.8/(*p* = 1.000)	−38.3/(*p* = 0.057)	−17.8/(*p* = 0.150)
**Chromosome 7 gain (+7)**							
Yes	3 (25.0)	5 (33.3)	8 (29.6)	13 (50.0)	+16.7/(*p* = 0.300)	+8.3/(*p* = 0.696)	+20.4/(*p* = 0.130)
No	1 (8.3)	5 (33.3)	6 (22.2)	4 (15.4)	−17.9/(*p* = 0.248)	+25.0/(*p* = 0.182)	−6.8/(*p* = 0.728)
Not tested	8 (66.7)	5 (33.3)	13 (48.1)	9 (34.6)	+1.3/(*p* = 0.934)	−33.4/(*p* = 0.085)	−13.5/(*p* = 0.318)
**Chromosome 10 loss (−10)**							
Yes	4 (33.3)	8 (53.3)	12 (44.4)	18 (69.2)	+15.9/(*p* = 0.309)	+20.0/(*p* = 0.299)	+24.8/(*p* = 0.069)
No	1 (8.3)	3 (20.0)	4 (14.8)	3 (11.5)	−8.5/(*p* = 0.651)	+11.7/(*p* = 0.605)	−3.3/(*p* = 1.000)
Not tested	7 (58.3)	4 (26.7)	11 (40.7)	5 (19.2)	−7.5/(*p* = 0.701)	−31.6/(*p* = 0.130)	−21.5/(*p* = 0.088)
**EGFR amplification**							
Yes	4 (33.3)	7 (46.7)	11 (40.7)	11 (42.3)	−4.4/(*p* = 0.786)	+13.4/(*p* = 0.696)	+1.6/(*p* = 0.908)
No	0 (0.0)	3 (20.0)	3 (11.1)	6 (23.1)	+3.1/(*p* = 1.000)	+20.0/(*p* = 0.231)	+12.0/(*p* = 0.293)
Not tested	8 (66.7)	5 (33.3)	13 (48.1)	9 (34.6)	+1.3/(*p* = 0.934)	−33.4/(*p* = 0.085)	−13.5/(*p* = 0.318)
**WHO CNS5 diagnosis**							
Glioblastoma, IDH-wildtype, WHO grade 4	4 (33.3)	9 (60.0)	13 (48.1)	20 (76.9)	+16.9/(*p* = 0.300)	+26.7/(*p* = 0.168)	+28.8/(*p* = 0.031)
Glioblastoma, WHO grade 4	7 (58.3)	3 (20.0)	10 (37.0)	4 (15.4)	−4.6/(*p* = 0.693)	−38.3/(*p* = 0.057)	−21.6/(*p* = 0.074)
Astrocytoma, IDH-mutant, WHO grade 4	1 (8.3)	3 (20.0)	4 (14.8)	2 (7.7)	−12.3/(*p* = 0.336)	+11.7/(*p* = 0.605)	−7.1/(*p* = 0.669)
**Performance status**							
KPS at baseline, median (range)	90 (80–90)	90 (60–90)	90 (60–90)	90 (40–100)	0.0/(*p* = 0.375)	0.0/(*p* = 0.440)	0.0/(*p* = 0.501)
**KPS at baseline (category)**							
90–100	10 (83.3)	11 (73.3)	21 (77.8)	20 (76.9)	+3.6/(*p* = 1.000)	−10.0/(*p* = 0.662)	−0.9/(*p* = 0.941)
70–80	2 (16.7)	3 (20.0)	5 (18.5)	5 (19.2)	−0.8/(*p* = 1.000)	+3.3/(*p* = 1.000)	+0.7/(*p* = 1.000)
≤60	0 (0.0)	1 (6.7)	1 (3.7)	1 (3.8)	−2.9/(*p* = 1.000)	+6.7/(*p* = 1.000)	+0.1/(*p* = 1.000)

Data are presented as the median (IQR), mean ± SD or *n* (%). Continuous variables were compared with the Mann–Whitney U test (normality assessed by the Shapiro–Wilk test in the two groups being compared; Student’s *t*-test was used when both groups had *p* ≥ 0.05, with Welch’s correction when variances were unequal by Levene’s test) and categorical variables with the Pearson χ^2^ test or, for 2 × 2 tables with any expected count < 5, the Fisher exact test. Within variables with more than two categories, each category was compared separately (category present vs. absent). Differences are expressed as the difference in means or medians, as applicable, for continuous variables and as the difference in rates (percentage points) for categorical variables, and were computed from the values reported in the table; for variables summarized by the median (IQR), *p* values are derived from rank-based tests and may be statistically significant even when the difference in medians is zero. The pooled control column combines the non-overlap-period and overlap-period TMZ + Bev cohorts (*n* = 27). Comparison c is not adjusted for treatment era and therefore partly reflects the between-period differences quantified in column b. Percentages are column percentages and may not sum to 100 because of rounding. All *p* values are two-sided and were not adjusted for multiplicity. a Between-group difference = overlap-period VM26 + TMZ + Bev minus overlap-period TMZ + Bev. b Across-period change = overlap-period TMZ + Bev minus non-overlap-period TMZ + Bev, i.e., the same regimen compared across the two periods, which quantifies the effect of treatment era. The non-overlap period contained no patients treated with VM26 + TMZ + Bev (teniposide only became available again in China after 2022), so no between-group comparison is possible in that period; it is reported as a historical reference cohort only. c VM26 + TMZ + Bev vs. pooled control = overlap-period VM26 + TMZ + Bev minus the pooled TMZ + Bev control group (non-overlap and overlap periods combined, *n* = 27). This contrast is not adjusted for treatment era and therefore partly reflects the between-period differences quantified in column b. d Radiotherapy fractions were recorded only for patients who received radiotherapy as part of initial treatment (*n* = 7, 12 and 23 in the non-overlap-period TMZ + Bev, overlap-period TMZ + Bev and overlap-period VM26 + TMZ + Bev groups, respectively, and 19 in the pooled control group). e Molecular testing was not performed in 14 patients (*n* = 7, 3 and 4 in the three cohorts, and 10 in the pooled control group); these patients are shown as “Not tested”, and all percentages use the full cohort size as the denominator. f No 1p/19q codeletion was detected in the tested patients. Abbreviations: Bev, bevacizumab; CDKN2A/B, cyclin-dependent kinase inhibitor 2A/B; EGFR, epidermal growth factor receptor; IDH, isocitrate dehydrogenase; IQR, interquartile range; KPS, Karnofsky performance status; MGMT, O^6^-methylguanine-DNA methyltransferase; RT, radiotherapy; SD, standard deviation; TERT, telomerase reverse transcriptase; TMZ, temozolomide; VM26, teniposide; WHO CNS5, WHO Classification of Tumors of the Central Nervous System, 5th edition.

**Table 2:** Kaplan–Meier estimates of progression-free and overall survival by treatment arm, with log-rank tests.

Endpoint	Treatment Arm	*n*	Events, *n* (%)	Censored, *n*	Median (95% CI), Months	6-Month Survival, % (95% CI)	12-Month Survival, % (95% CI)	Log-Rank χ^2^	*p*
**Progression-free survival**								**4.358**	**0.037**
	VM26 + TMZ + Bev	26	15 (57.7)	11	7.8 (4.9–10.9)	65.0 (43.4–80.0)	24.4 (6.8–47.6)		
	TMZ + Bev	27	23 (85.2)	4	5.1 (4.4–6.8)	48.1 (28.7–65.2)	5.6 (0.4–21.9)		
**Overall survival**								**4.147**	**0.042**
	VM26 + TMZ + Bev	26	14 (53.8)	12	12.7 (8.4–15.4)	79.1 (56.7–90.8)	50.7 (26.9–70.4)		
	TMZ + Bev	27	21 (77.8)	6	8.5 (7.3–9.7)	85.0 (64.9–94.1)	5.0 (0.4–20.6)		

CI, confidence interval; PFS, progression-free survival; OS, overall survival. Survival probabilities were estimated by using the Kaplan–Meier method and are reported at 6 and 12 months (180 and 365 days); 95% confidence intervals were calculated from the Greenwood variance with a log–log transformation. Median survival times are expressed in months, calculated as days divided by 30.4375. An event was defined as progression or death for PFS and as death for OS; all remaining patients were censored at the date of last follow-up. The time origin was the date on which chemotherapy was first initiated after recurrence for both endpoints. The log-rank test compares the two treatment arms within each endpoint; the test statistic has one degree of freedom. *p* values are two-sided and were not adjusted for multiple comparisons, in keeping with the exploratory nature of the study. Both endpoints favored the VM-26-containing regimen. These comparisons are not adjusted for treatment era; the era-matched and cross-era survival analyses are reported in Table S8.

**Table 3:** Univariable and multivariable Cox regression analysis for progression-free survival.

Variable	Univariable HR (95% CI)	*p*	Multivariable HR (95% CI)	*p*
Treatment (VM26 + TMZ + Bev vs. TMZ + Bev) *	0.50 (0.25–0.97)	0.041	0.42 (0.19–0.95)	0.037
Age, per year	1.01 (0.98–1.03)	0.541	—	—
Male sex	0.49 (0.25–0.95)	0.033	—	—
Body-mass index, per kg/m^2^	0.97 (0.89–1.05)	0.460	—	—
KPS ≥ 90 *	1.05 (0.48–2.30)	0.911	0.91 (0.41–2.03)	0.813
Glioblastoma, IDH-wildtype *	0.92 (0.48–1.78)	0.812	1.42 (0.67–3.01)	0.361
Multiple lesions at recurrence	0.80 (0.37–1.69)	0.552	—	—
Recurrence surgery before chemotherapy *	1.38 (0.68–2.79)	0.378	1.07 (0.50–2.27)	0.866
Gross total resection at initial surgery	1.10 (0.56–2.16)	0.780	—	—
Eloquent region involvement	0.82 (0.39–1.75)	0.612	—	—
Any comorbidity	0.98 (0.52–1.86)	0.951	—	—
log(Time to first recurrence)	0.92 (0.63–1.36)	0.688	—	—
Corticosteroid use	1.13 (0.55–2.34)	0.738	—	—

* Included in the multivariable model (treatment plus three pre-specified covariates). The multivariable model comprised treatment, KPS ≥ 90, glioblastoma IDH-wildtype and recurrence surgery completed before chemotherapy; concordance index = 0.588. HR, hazard ratio; CI, confidence interval. Univariable estimates are from separate models, each containing a single variable. Exact *p* values are reported to three decimal places. These models are not adjusted for treatment era; the era-matched analyses are reported in Table S8. Proportional hazards assumption (scaled Schoenfeld residuals, multivariable model): Treatment *p* = 0.173; KPS ≥ 90 *p* = 0.030; Glioblastoma, IDH-wildtype *p* = 0.731; Recurrence surgery before chemotherapy *p* = 0.748.

**Table 4:** Univariable and multivariable Cox regression analysis for overall survival.

Variable	Univariable HR (95% CI)	*p*	Multivariable HR (95% CI)	*p*
Treatment (VM26 + TMZ + Bev vs. TMZ + Bev) *	0.47 (0.23–0.98)	0.045	0.32 (0.12–0.85)	0.022
Age, per year	1.00 (0.98–1.03)	0.843	—	—
Male sex	0.62 (0.31–1.26)	0.188	—	—
Body mass index, per kg/m^2^	0.98 (0.91–1.07)	0.705	—	—
KPS ≥ 90 *	0.92 (0.41–2.08)	0.846	0.65 (0.27–1.54)	0.325
Glioblastoma, IDH-wildtype *	0.95 (0.47–1.89)	0.875	1.73 (0.75–3.97)	0.195
Multiple lesions at recurrence	1.97 (0.91–4.25)	0.084	—	—
Recurrence surgery before chemotherapy *	1.43 (0.69–2.95)	0.340	0.94 (0.41–2.15)	0.876
Gross total resection at initial surgery	0.88 (0.43–1.81)	0.726	—	—
Eloquent region involvement	1.39 (0.64–3.01)	0.402	—	—
Any comorbidity	1.10 (0.56–2.17)	0.788	—	—
Log (Time to first recurrence)	0.95 (0.63–1.42)	0.797	—	—
Corticosteroid use	1.27 (0.57–2.80)	0.559	—	—

* Included in the multivariable model (treatment plus three pre-specified covariates). The multivariable model comprised treatment, KPS ≥ 90, glioblastoma IDH-wildtype and recurrence surgery completed before chemotherapy; concordance index = 0.598. HR, hazard ratio; CI, confidence interval. Univariable estimates are from separate models, each containing a single variable. Exact *p* values are reported to three decimal places. These models are not adjusted for treatment era; the era-matched analyses are reported in Table S8. Proportional hazards assumption (scaled Schoenfeld residuals, multivariable model): Treatment *p* = 0.293; KPS ≥ 90 *p* = 0.280; Glioblastoma, IDH-wildtype *p* = 0.419; Recurrence surgery before chemotherapy *p* = 0.332.

**Table 5:** Comparison of efficacy endpoints between the overlap and non-overlap periods, and against the pooled TMZ + Bev control.

Indicator	Non-Overlap Period (2020–2022) TMZ + Bev (*n* = 12)	Overlap Period (2023–2026) TMZ + Bev (*n* = 15)	Overlap Period (2023–2026) VM26 + TMZ + Bev (*n* = 26)	Between-Group Difference a (*p* Value)	Across- Period Change b (*p* Value)	Pooled Control (2020–2026) TMZ + Bev (*n* = 27)	VM26 + TMZ + Bev vs. Pooled Control c (*p* Value)
Best response: PR	2 (16.7)	2 (13.3)	7 (26.9)	+13.6 (*p* = 0.445)	−3.3 (*p* = 1.000)	4 (14.8)	+12.1 (*p* = 0.327)
Best response: SD	6 (50.0)	11 (73.3)	14 (53.8)	−19.5 (*p* = 0.218)	+23.3 (*p* = 0.257)	17 (63.0)	−9.1 (*p* = 0.583)
Best response: PD	4 (33.3)	2 (13.3)	5 (19.2)	+5.9 (*p* = 1.000)	−20.0 (*p* = 0.357)	6 (22.2)	−3.0 (*p* = 1.000)
Objective response rate (PR)	2 (16.7)	2 (13.3)	7 (26.9)	+13.6 (*p* = 0.445)	−3.3 (*p* = 1.000)	4 (14.8)	+12.1 (*p* = 0.327)
Disease control rate (PR + SD)	8 (66.7)	13 (86.7)	21 (80.8)	−5.9 (*p* = 1.000)	+20.0 (*p* = 0.357)	21 (77.8)	+3.0 (*p* = 1.000)
Early progression (PFS < 2 months)	0 (0.0)	1 (6.7)	4 (15.4)	+8.7 (*p* = 0.636)	+6.7 (*p* = 1.000)	1 (3.7)	+11.7 (*p* = 0.192)
Interval from index date to response assessment, months	4.93 (4.36–6.82)	5.22 (3.33–6.93)	4.90 (2.88–5.89)	−0.33 (*p* = 0.507)	+0.30 (*p* = 0.788)	5.13 (4.06–6.87)	−0.23 (*p* = 0.328)

Data are presented as the median (IQR) or *n* (%). Continuous variables were compared with the Mann–Whitney U test (normality assessed by the Shapiro–Wilk test; Student’s *t*-test was used when both groups had *p* ≥ 0.05). Categorical variables were compared with the Fisher exact test (2 × 2 tables with any expected count < 5) or the Pearson χ^2^ test, except for the pooled control comparison, in which the Fisher exact test was applied to all 2 × 2 comparisons (footnote c); for r × c tables with any expected count < 5, a Monte-Carlo exact test was used (20,000 replicates, seed = 42). Efficacy was assessed with an intention-to-treat denominator: patients who progressed or died before the assessment window were counted as PD. All *p* values are two-sided and were not adjusted for multiplicity. a Between-group difference = overlap-period VM26 + TMZ + Bev minus overlap-period TMZ + Bev. Differences are expressed as the difference in medians for continuous variables and as the difference in rates (percentage points) for categorical variables. b Across-period change = overlap-period TMZ + Bev minus non-overlap-period TMZ + Bev, i.e., the same regimen compared across the two periods, which quantifies the effect of treatment era. The non-overlap period contained no patients treated with VM26 + TMZ + Bev (teniposide only became available again in China after 2022), so no between-group comparison is possible in that period; it is reported as a historical reference cohort only. c Pooled control comparison = overlap-period VM26 + TMZ + Bev minus the pooled TMZ + Bev group (patients from both periods combined, 2020–2026; *n* = 27). Differences are expressed as the difference in medians for continuous variables and as the difference in rates (percentage points) for categorical variables. Categorical variables were compared with the Fisher exact test, consistent with the primary efficacy analysis, and continuous variables with the Mann–Whitney U test. Abbreviations: Bev, bevacizumab; DCR, disease control rate; IQR, interquartile range; ITT, intention-to-treat; ORR, objective response rate; PD, progressive disease; PFS, progression-free survival; PR, partial response; SD, stable disease; TMZ, temozolomide; VM26, teniposide.

**Table 6:** Treatment-related adverse events.

Endpoint	TMZ + Bev 2020–2022 (*n* = 12)	TMZ + Bev 2023–2026 (*n* = 15)	VM26 + TMZ + Bev 2023–2026 (*n* = 26)	Between-Group Difference a	Across-Period Change b	TMZ + Bev, Pooled 2020–2026 (*n* = 27)	VM26 vs. Pooled Control c
**Safety endpoints (ITT denominator)**							
Any AE, any grade	3 (25.0)	4 (26.7)	12 (46.2)	+19.5 (*p* = 0.322)	+1.7 (*p* = 1.000)	7 (25.9)	+20.2 (*p* = 0.158)
Grade ≥ 3 AE	1 (8.3)	1 (6.7)	3 (11.5)	+4.9 (*p* = 1.000)	−1.7 (*p* = 1.000)	2 (7.4)	+4.1 (*p* = 0.669)
Any hematologic AE, any grade	3 (25.0)	4 (26.7)	11 (42.3)	+15.6 (*p* = 0.502)	+1.7 (*p* = 1.000)	7 (25.9)	+16.4 (*p* = 0.254)
Any non-hematologic AE, any grade	1 (8.3)	1 (6.7)	6 (23.1)	+16.4 (*p* = 0.232)	−1.7 (*p* = 1.000)	2 (7.4)	+15.7 (*p* = 0.142)
**Hematologic toxicity**							
Leukopenia	1 (8.3)	0 (0.0)	7 (26.9)	+26.9 (*p* = 0.035)	−8.3 (*p* = 0.444)	1 (3.7)	+23.2 (*p* = 0.024)
Neutropenia	1 (8.3)	0 (0.0)	7 (26.9)	+26.9 (*p* = 0.035)	−8.3 (*p* = 0.444)	1 (3.7)	+23.2 (*p* = 0.024)
Thrombocytopenia	0 (0.0)	0 (0.0)	1 (3.8)	+3.8 (*p* = 1.000)	0.0 (*p* = 1.000)	0 (0.0)	+3.8 (*p* = 0.491)
Anemia	0 (0.0)	0 (0.0)	1 (3.8)	+3.8 (*p* = 1.000)	0.0 (*p* = 1.000)	0 (0.0)	+3.8 (*p* = 0.491)
Lymphopenia	2 (16.7)	4 (26.7)	6 (23.1)	−3.6 (*p* = 1.000)	+10.0 (*p* = 0.662)	6 (22.2)	+0.9 (*p* = 1.000)
**Non-hematologic toxicity**							
Fatigue	0 (0.0)	0 (0.0)	2 (7.7)	+7.7 (*p* = 0.524)	0.0 (*p* = 1.000)	0 (0.0)	+7.7 (*p* = 0.236)
Hypertension	0 (0.0)	0 (0.0)	1 (3.8)	+3.8 (*p* = 1.000)	0.0 (*p* = 1.000)	0 (0.0)	+3.8 (*p* = 0.491)
Infection	0 (0.0)	0 (0.0)	1 (3.8)	+3.8 (*p* = 1.000)	0.0 (*p* = 1.000)	0 (0.0)	+3.8 (*p* = 0.491)
Nausea	0 (0.0)	0 (0.0)	3 (11.5)	+11.5 (*p* = 0.287)	0.0 (*p* = 1.000)	0 (0.0)	+11.5 (*p* = 0.111)
Vomiting	1 (8.3)	1 (6.7)	2 (7.7)	+1.0 (*p* = 1.000)	−1.7 (*p* = 1.000)	2 (7.4)	+0.3 (*p* = 1.000)
**Treatment modification**							
Dose reduction due to toxicity	1 (8.3)	1 (6.7)	3 (11.5)	+4.9 (*p* = 1.000)	−1.7 (*p* = 1.000)	2 (7.4)	+4.1 (*p* = 0.669)
Treatment discontinuation due to AE	0 (0.0)	1 (6.7)	3 (11.5)	+4.9 (*p* = 1.000)	+6.7 (*p* = 1.000)	1 (3.7)	+7.8 (*p* = 0.351)
Highest CTCAE grade	0.0 (0.0–0.2)	0.0 (0.0–0.5)	0.0 (0.0–1.0)	0.0 (*p* = 0.300)	0.0 (*p* = 0.874)	0.0 (0.0–0.5)	0.0 (*p* = 0.154)

Data are presented as the median (IQR) or *n* (%). Continuous variables were compared with the Mann–Whitney U test; Student’s *t*-test was used when the Shapiro–Wilk test indicated normality. Categorical variables were compared with the Fisher exact test. Adverse events were graded according to CTCAE version 5.0 and are summarized as the worst grade recorded for each patient during salvage therapy; grade 0 denotes the absence of the event. All proportions use the ITT denominator of each group (*n* = 12, *n* = 15 and *n* = 26, respectively). Continuous variables are reported to one decimal place, percentages to one decimal place, and *p* values to three decimal places. Values in the two right-hand columns are differences (percentage points for categorical variables and differences in medians for continuous variables), with two-sided *p* values in parentheses. a Between-group difference = overlap-period VM26 + TMZ + Bev minus overlap-period TMZ + Bev. Both groups were treated during the same calendar period (2023–2026), so this contrast estimates the effect of adding teniposide while holding the treatment era constant. Differences are expressed as the difference in medians for continuous variables and as the difference in percentages for categorical variables. *p* values are two-sided and were calculated with the Mann–Whitney U test (continuous) or the Fisher exact test (categorical). b Across-period change = overlap-period TMZ + Bev minus non-overlap-period TMZ + Bev, i.e., the same regimen compared across the two periods, which quantifies the effect of treatment era. The non-overlap period comprises patients treated before teniposide became available at our center (2020–2022), so these patients had no access to the study regimen and serve as a historical reference cohort only. c Pooled control analysis = overlap-period VM26 + TMZ + Bev minus all patients treated with TMZ + Bev irrespective of period (non-overlap and overlap periods combined; *n* = 27). This secondary analysis uses the entire control cohort rather than the era-matched control group. Differences are expressed as the difference in medians for continuous variables and as the difference in percentages for categorical variables, and *p* values are two-sided (Mann–Whitney U test for continuous variables; Fisher exact test for categorical variables). Abbreviations: AE, adverse event; Bev, bevacizumab; CTCAE, Common Terminology Criteria for Adverse Events; IQR, interquartile range; ITT, intention-to-treat; TMZ, temozolomide; VM26, teniposide.

## Data Availability

The de-identified patient-level dataset and the supplementary tables supporting the conclusions of this study (Tables S1–S10) are provided as Supplementary Materials. Additional information is available from the corresponding author on reasonable request.

## Supplementary Materials

The following supporting information can be downloaded at: https://www.mdpi.com/article/10.3390/jcm15207827/s1. Table S1. Treatment effect across the eleven pre-specified analysis strategies and the additional model requested during review. Table S2. Propensity-score model (logistic regression for assignment to VM26 + TMZ + Bev). Table S3. Proportional-hazards assumption assessed by scaled Schoenfeld residuals. Table S4. Handling of the KPS-related proportional-hazards violation: effect of each approach on the treatment estimate. Table S5. Complete-case analysis restricted to the 39 patients who underwent molecular testing. Table S6. Baseline characteristics of patients who underwent molecular testing versus those who did not (assessment of selection bias). Table S7. Multicollinearity diagnostics among the molecular and clinical covariates. Table S8. Survival analyses accounting for treatment era. Table S9. Clinical considerations recorded at the time of treatment selection, by treatment group. Table S10. Sensitivity analysis restricted to glioblastoma, IDH-wildtype, WHO grade 4. Supplementary Material S1 (Supplementary Material S1_clinical data.xlsx). De-identified patient-level dataset. Supplementary Material S2 (Supplementary Material S2_consent form.pdf). Scanned copies of the written informed consent forms.
