# Supplementary material for: The Efficacy and Safety of Adding Teniposide to Temozolomide Plus Bevacizumab in Recurrent Grade 4 Glioma: A Real-World Exploratory Single-Center Study
Source: J Clin Med. 2026 Oct 10;15(20):7827. doi: 10.3390/jcm15207827 (PMC13705379; doi:10.3390/jcm15207827)
Supplement: Supplementary file 1 [file jcm-15-07827-s001.zip › Supplementary Material S2_consent form.pdf]

中国医学科学院肿瘤医院
Cancer Hospital Chinese Academy of Medical Sciences

化疗知情同意书

姓名：[redacted] 病案号：[redacted]

姓名：[redacted] 性别：男 年龄：[redacted] 科室：神经外科1

病区：神经外科病房 床位号：[redacted]

尊敬的患者：

根据您的病情，经过慎重考虑，医生建议您接受此次内科药物抗肿瘤治疗。现将您的病情、拟行治疗方案及可能发生的风险说明如下。请您详细阅读，并与您的医生、家人和朋友充分讨论，最终签署您的意见。

临床诊断：脑恶性肿瘤(脑胶质母细胞瘤术后、放化疗后多发转移，12周期挽救治疗后)，脊膜继发恶性肿瘤(颈胸腰椎管内多发转移)，中性粒细胞减少症，白细胞减少，呕吐，恶心，疼痛(重度)，恶性肿瘤术后化疗，恶性肿瘤靶向治疗，中枢神经损伤，肝功能损伤

分期：IV

内科药物抗肿瘤治疗类别：

内科药物抗肿瘤治疗主要包括化学治疗、内分泌治疗、靶向治疗和生物免疫治疗等。您拟行的治疗属于：

化学治疗

拟行的具体治疗方案：替尼泊苷 50mg d1-d3+替莫唑胺400mg d1-d5+贝伐单抗 400mg+鞘内注射 塞替派 10mg

替代方案：

我们认为，以上方案可能比较适合您，但其他的药物或治疗手段也可能适合您，包括但不限于：

其他的药物治疗

您可以就您所关心的其他治疗方案的问题详细咨询您的医生，充分比较后再做出决定。您可以自主选择您认可的其他治疗方案，您不会因为您的选择受到不公正的对待。在治疗过程中，可能会根据病情变化和治疗反应调整方案。

拟行抗肿瘤治疗方案可能发生的情况及风险：

内科的药物抗肿瘤治疗手段在在抑制和杀死肿瘤细胞的同时，对人体某些正常组织细胞也有一定的毒性，因此治疗过程中存在一定的风险。特此郑重向您说明拟行的治疗方案可能发生的不良反应和风险，包括但不限于以下内容：

拟行化疗方案可能出现的副作用包括，但不仅限于：

全身一般反应：疲乏、虚弱、发热、头疼、过敏反应、肌肉关节疼痛、腹痛等；

药物输注相关/过敏反应：如寒战、发热、皮疹、皮肤潮红、瘙痒、剥脱性皮炎、低血压、血管性水肿、支气管痉挛、高血压、休克等；

消化道反应：食欲减退、恶心、呕吐、腹泻、便秘、口腔溃疡、胃肠黏膜炎和味觉改变等；

骨髓抑制：白细胞减少、中性粒细胞减少、血小板减少、贫血、再生障碍性贫血；

肝肾毒性：如转氨酶、胆红素、碱性磷酸酶、转肽酶异常、肾功能不全、蛋白尿等；

代谢异常：血脂、血糖、电解质紊乱、淀粉酶升高、血蛋白质水平异常；

神经毒性：如头晕、头疼、手足麻木、感觉异常、视物模糊、听力障碍、嗜睡、失眠、共济失调、精神异常等；严重者可以引起肠麻痹、运动障碍；

眼毒性：如结膜炎、眼睛功能异常、视力敏度减低、视野异常和视神经炎等；

心脏毒性：如胸闷、心悸、心律失常、心功能下降、心肌缺血、心电图异常等

肺毒性：咳嗽、胸痛、呼吸困难、非感染性肺炎等；

输注部位反应：部分化疗药物局部刺激性较强，可引起静脉炎；外渗或外漏后可能造成局部组织损伤，严重者可引起蜂窝织炎、组织坏死和纤维化；

化疗知情同意书

姓名：[redacted]　　　　　　　　　　　　　　　　病案号：[redacted]

皮肤毒性：脱发、手足皮肤反应、皮肤黏膜色素沉着、皮疹、瘙痒、指甲改变、甲沟炎等。

此外，化疗有可能导致胎儿畸形或死亡，所以在化疗期间必须正规避孕。多数化疗副作用在开始化疗后短期内出现，但也有一些副作用可能在化疗结束较长时间后出现。例如，化疗可能造成生育方面的困难，因此必要时可以在化疗前冻存精子或卵子。其他脏器也可能出现远期毒性，如心脏功能减退。

化疗的副作用多数在化疗结束后可以恢复，但也有一些不能完全恢复，甚至造成永久的损害，如肺纤维化，心功能不全等。

在治疗中或治疗后，还可能出现其他少见或罕见的副作用以及以上未提及或难以预料的副作用。不良反应的种类和严重程度因药物类型、个体差异而不同，严重者可能危及生命。

我们将严格执行抗肿瘤药物治疗操作规范，在治疗过程中严密监测不良反应。一旦发生意外或并发症，我们将积极采取相应的处理和抢救措施。但由于目前医疗水平仍然存在一定的局限性及个体体质的差异，治疗过程中仍然可能出现严重的组织器官损害，甚至危及生命。出现不良反应或意外情况时采取的相关救治措施可能造成病情迁延、医疗费用的增加、需要再次或数次手术等情况的出现。

内科药物治疗作为肿瘤治疗的重要手段广泛应用于临床，并对多种肿瘤具有良好的疗效。内科药物治疗配合手术、放疗、生物靶向和免疫治疗等手段能够进一步提高多种肿瘤的治疗效果，部分患者经过药物治疗可以获得治愈，更多的晚期肿瘤病人经过药物治疗可以延长生存时间或改善生活质量。但是，不是所有肿瘤都对药物治疗敏感。因此，药物治疗有可能有效，但也可能完全无效。

患者自身存在的高危因素：

一般情况差

因患者存在以上合并情况，抗肿瘤药物治疗风险可能会增加，且会增加治疗过程中不良反应处理的复杂性和困难。治疗过程中也有可能出现相关病情的加重、心脑血管意外、甚至死亡。

患者有义务如实告知既往病史、治疗史及用药史，否则可能对于本次治疗造成严重不良后果。

知情同意申明：

医师已经向我解释过我的病情、拟采取抗肿瘤治疗目的、可能产生的风险、及我自身可能存在的高危因素等情况，我也就我关心的问题向医生进行了详细询问并得到了满意答复。我知道我有权拒绝或放弃抗肿瘤药物治疗，并不会因此遭受不公平的对待。

医师就我病情可以采取的其他治疗方式的效果和风险向我进行了告知，我也就我关心的问题向医生进行了详细询问并得到满意答复。

中国医学科学院肿瘤医院是一家三级甲等肿瘤专科医院，所从事的医疗活动严格遵循国家有关的法律、法规和诊疗护理的规范要求。我同意在这家医院诊疗过程中引起的任何争议将按照国家有关的法律、法规所提供的途径解决。

经过认真考虑，我同意进行抗肿瘤药物治疗。

临床资料和生物样本用于科学研究的告知和知情同意

中国医学科学院肿瘤医院是一所集肿瘤预防、治疗、研究于一体的国家级肿瘤专科医学中心，临床治疗与医学研究相结合是我中心的特色之一。在不影响正常诊疗的情况下，如果得到您的同意，您的临床资料（如病情记录）和生物样本（例如诊断、检测和治疗后留存的多余血标本和组织标本）有可能用于合理的医学科学研究。这些研究可能有助于改进目前您所患疾病的治疗方法。我们承诺这些研究不会给您的治疗带来不利影响，并且不会侵犯您的隐私权和其他正当权益。您目前所患疾病的最佳治疗也来自于以往患者对科学研究的参与，我们感谢您对医学发展做出的贡献。

您可以选择同意或不同意将您的临床资料和生物样本用于科学研究。您无论同意或不同意，都不会影响您的治疗和正当权益。

知情同意申明：

中国医学科学院肿瘤医院
Cancer Hospital Chinese Academyof Medical Sciences

化疗知情同意书

姓名：[redacted]　　　　病案号：[redacted]

患者签名：[redacted]　　　　日期：2025年06月11日

（应由患者本人签名，如果患者本人无法签名，可由患者法定代理人、近亲属及关系人签名，同时注明与患者关系，并附[redacted]）

医师签名：[redacted]　　　　日期：2025年06月11日

（应由与患[redacted]行谈话的医师，包括手术者、手术者委托的参与本手术的医师签名。）
