# Supplementary material for: The Efficacy and Safety of Adding Teniposide to Temozolomide Plus Bevacizumab in Recurrent Grade 4 Glioma: A Real-World Exploratory Single-Center Study
Source: J Clin Med. 2026 Oct 10;15(20):7827. doi: 10.3390/jcm15207827 (PMC13705379; doi:10.3390/jcm15207827)
Supplement: Supplementary file 1 [file jcm-15-07827-s001.zip › Table_S10_IDH_wildtype_sensitivity_analysis.pdf]

Table S10. Sensitivity analysis restricted to glioblastoma, IDH-wildtype, WHO grade 4.

Analysis	Endpoint	n	Median, months (95% CI) † VM26 triplet	Median, months (95% CI) † control	HR (95% CI)	P
Glioblastoma, IDH-wildtype, WHO grade 4 (molecularly defined subset) ‡	PFS	20 vs 13	7.8 (3.9–NR)	4.8 (3.8–6.7)	0.35 (0.14–0.89)	0.021 §
	OS	20 vs 13	12.7 (8.4–15.4)	7.8 (6.8–10.0)	0.32 (0.12–0.82)	0.012 §
Glioblastoma, IDH-wildtype, WHO grade 4, concurrently treated cohorts only (2023–2026) ‡	PFS	20 vs 9	7.8 (3.9–NR)	5.0 (1.3–7.2)	0.42 (0.15–1.17)	0.087 §
	OS	20 vs 9	12.7 (8.4–15.4)	7.8 (2.7–10.3)	0.39 (0.13–1.13)	0.073 §
Full cohort (all diffuse gliomas, WHO CNS5 grade 4), for reference ¶	PFS	26 vs 27	7.8 (4.9–10.9)	5.1 (4.4–6.8)	0.50 (0.25–0.97)	0.037 §
	OS	26 vs 27	12.7 (8.4–15.4)	8.5 (7.3–9.7)	0.47 (0.23–0.98)	0.042 §

HR, hazard ratio; CI, confidence interval; PFS, progression-free survival; OS, overall survival; NR, not reached; CNS5, WHO Classification of Tumours of the Central Nervous System, 5th edition; VM26, teniposide; TMZ, temozolomide; Bev, bevacizumab. All hazard ratios compare the VM-26-containing triplet with the control group; an HR below 1 indicates benefit for the triplet. † Median survival was estimated by the Kaplan–Meier method and the 95% CI was derived from the Greenwood variance estimator with a log–log transformation; median survival times are expressed in months, calculated as days divided by 30.4375. ‡ Patients were classified according to the integrated histological and molecular diagnosis recorded in the medical report; the 6 patients with astrocytoma, IDH-mutant, WHO grade 4 and the 14 patients whose tumors were not tested are excluded from these subsets. § Two-sided log-rank P value. The corresponding Cox P values were 0.027 (PFS) and 0.017 (OS) in the molecularly defined subset, 0.096 and 0.083 in the concurrently treated subset, and 0.041 and 0.045 in the full cohort. ¶ The full-cohort estimates reproduce the primary analysis (Table 2) and are shown for reference only. This analysis was performed in response to peer review and is not counted among the 11 pre-specified strategies in Table S1. All analyses are on an intention-to-treat basis and are not adjusted for multiplicity.
