# Supplementary material for: The Efficacy and Safety of Adding Teniposide to Temozolomide Plus Bevacizumab in Recurrent Grade 4 Glioma: A Real-World Exploratory Single-Center Study
Source: J Clin Med. 2026 Oct 10;15(20):7827. doi: 10.3390/jcm15207827 (PMC13705379; doi:10.3390/jcm15207827)
Supplement: Supplementary file 1 [file jcm-15-07827-s001.zip › Table_S1_Treatment_effect_across_analysis_strategies.pdf]

Table S1. Treatment effect across the eleven pre-specified analysis strategies and the additional model requested during review.

Analysis strategy	n	PFS HR (95% CI)	P	OS HR (95% CI)	P
Univariable Cox	53	0.50 (0.25–0.97)	0.041	0.47 (0.23–0.98)	0.045
Multivariable Cox (primary model)	53	0.42 (0.19–0.95)	0.037	0.32 (0.12–0.85)	0.022
Multivariable Cox + multiple lesions at recurrence †	53	0.42 (0.18–0.99)	0.048	0.26 (0.09–0.70)	0.008
Propensity score as a covariate	53	0.63 (0.27–1.49)	0.291	0.39 (0.14–1.08)	0.069
Inverse probability of treatment weighting	53	0.60 (0.29–1.21)	0.155	0.52 (0.23–1.16)	0.110
Propensity-score matching + stratified Cox (13 pairs)	26	0.71 (0.23–2.25)	0.566	0.33 (0.07–1.65)	0.179
Cox model stratified by KPS	53	0.42 (0.19–0.96)	0.041	0.35 (0.13–0.95)	0.039
Time-varying-coefficient model	53	0.41 (0.18–0.91)	0.028	0.35 (0.14–0.88)	0.026
Landmark analysis at 60 days	48 / 52	0.28 (0.11–0.69)	0.006	0.24 (0.09–0.67)	0.007
Landmark analysis at 90 days	45 / 51	0.25 (0.09–0.67)	0.006	0.29 (0.10–0.83)	0.021
Complete-case analysis (tested patients)	39	0.51 (0.20–1.29)	0.155	0.32 (0.10–1.02)	0.055
Complete-case analysis + MGMT adjustment	39	0.49 (0.20–1.19)	0.116	0.34 (0.13–0.87)	0.025

HR, hazard ratio; CI, confidence interval; KPS, Karnofsky Performance Status; MGMT, O6-methylguanine-DNA methyltransferase. All estimates compare VM26 + TMZ + Bev with TMZ+Bev; HR < 1 indicates benefit for the VM-26-containing regimen. For landmark analyses, the two n values refer to the PFS and OS populations after exclusion of events occurring before the landmark time. Every point estimate is below 1, i.e. the direction of effect is consistent across all strategies; the propensity-score-based analyses and the complete-case analysis did not reach statistical significance, which is attributable to the limited number of events (38 for PFS and 35 for OS). The three analyses that account for treatment era are reported separately in Table S8. † Added after the primary analysis in response to a reviewer's comment; this model is a sensitivity variant of the primary multivariable model, with multiple lesions at recurrence added as a fourth covariate. It is not counted among the eleven pre-specified strategies.
