# Supplementary material for: The Efficacy and Safety of Adding Teniposide to Temozolomide Plus Bevacizumab in Recurrent Grade 4 Glioma: A Real-World Exploratory Single-Center Study
Source: J Clin Med. 2026 Oct 10;15(20):7827. doi: 10.3390/jcm15207827 (PMC13705379; doi:10.3390/jcm15207827)
Supplement: Supplementary file 1 [file jcm-15-07827-s001.zip › Table_S2_Propensity_score_model.pdf]

Table S2. Propensity-score model (logistic regression for assignment to VM26 + TMZ + Bev).

Covariate	Coefficient	P
Age, per year	+0.004	0.8688
Male sex	+2.057	0.0166
Glioblastoma, IDH-wildtype	+2.247	0.0085
Multiple lesions at recurrence	+0.759	0.3136
Recurrence surgery before chemotherapy	−1.253	0.0945
Gross total resection at initial surgery	−0.444	0.5420
KPS ≥ 90	−0.383	0.6213
Any comorbidity	−0.583	0.4918

McFadden pseudo-R^2 = 0.233. The propensity score was used as (i) a single covariate, (ii) the basis of stabilized inverse-probability-of-treatment weights, and (iii) the basis of 1:1 nearest-neighbor matching (caliper = 0.2 × SD of the logit of the propensity score), which yielded 13 matched pairs.
