# Supplementary material for: The Efficacy and Safety of Adding Teniposide to Temozolomide Plus Bevacizumab in Recurrent Grade 4 Glioma: A Real-World Exploratory Single-Center Study
Source: J Clin Med. 2026 Oct 10;15(20):7827. doi: 10.3390/jcm15207827 (PMC13705379; doi:10.3390/jcm15207827)
Supplement: Supplementary file 1 [file jcm-15-07827-s001.zip › Table_S3_Proportional_hazards_assumption.pdf]

Table S3. Proportional-hazards assumption assessed by scaled Schoenfeld residuals.

Model	Variable	χ^2	P	Assessment
PFS primary model	Treatment (VM26 + TMZ + Bev vs TMZ+Bev)	1.859	0.173	Assumption met
	KPS ≥ 90	4.718	0.030	Borderline violation
	Glioblastoma, IDH-wildtype	0.118	0.731	Assumption met
	Recurrence surgery before chemotherapy	0.103	0.748	Assumption met
OS primary model	Treatment (VM26 + TMZ + Bev vs TMZ+Bev)	1.107	0.293	Assumption met
	KPS ≥ 90	1.169	0.280	Assumption met
	Glioblastoma, IDH-wildtype	0.654	0.419	Assumption met
	Recurrence surgery before chemotherapy	0.942	0.332	Assumption met

$P < 0.05$ indicates a violation of the proportional-hazards assumption. The treatment effect — the estimate of primary interest — satisfied the assumption in both models ($P = 0.173$ and $P = 0.293$). Only KPS ≥ 90 in the PFS model showed a violation. Its handling is described in Table S4; the KPS binary groups showed no association with outcome on log-rank testing ($P = 0.914$ for PFS and $P = 0.842$ for OS), and the treatment estimate was unchanged across all four remedial approaches, indicating a statistical artefact rather than a substantive violation.
