# Supplementary material for: The Efficacy and Safety of Adding Teniposide to Temozolomide Plus Bevacizumab in Recurrent Grade 4 Glioma: A Real-World Exploratory Single-Center Study
Source: J Clin Med. 2026 Oct 10;15(20):7827. doi: 10.3390/jcm15207827 (PMC13705379; doi:10.3390/jcm15207827)
Supplement: Supplementary file 1 [file jcm-15-07827-s001.zip › Table_S4_KPS_PH_violation_handling.pdf]

Table S4. Handling of the KPS-related proportional-hazards violation: effect of each approach on the treatment estimate.

Approach	PFS HR (95% CI)	P	OS HR (95% CI)	P
Primary model (KPS as a covariate)	0.42 (0.19–0.95)	0.037	0.32 (0.12–0.85)	0.022
Cox model stratified by KPS	0.42 (0.19–0.96)	0.041	0.35 (0.13–0.95)	0.039
Time-varying-coefficient model (KPS × ln t, log t centered on its median)	0.41 (0.18–0.91)	0.028	0.35 (0.14–0.88)	0.026
Landmark analysis at 60 days	0.28 (0.11–0.69)	0.006	0.24 (0.09–0.67)	0.007
Landmark analysis at 90 days	0.25 (0.09–0.67)	0.006	0.29 (0.10–0.83)	0.021

In the time-varying-coefficient model, log(t) was centered on its median before the interaction term was constructed; without this centring, the main effect of KPS and the interaction term are structurally collinear and the reported hazard ratio becomes numerically unstable (uncentered estimates: HR 596 for PFS and 53 for OS, with confidence intervals spanning three to five orders of magnitude). The centered model yields a treatment hazard ratio of 0.41 (95% CI 0.18–0.91) for PFS and 0.35 (0.14–0.88) for OS, in close agreement with the primary model. The time-varying hazard ratio for KPS itself is not clinically interpretable and is reported only to document the source of the apparent violation.
