# Supplementary material for: The Efficacy and Safety of Adding Teniposide to Temozolomide Plus Bevacizumab in Recurrent Grade 4 Glioma: A Real-World Exploratory Single-Center Study
Source: J Clin Med. 2026 Oct 10;15(20):7827. doi: 10.3390/jcm15207827 (PMC13705379; doi:10.3390/jcm15207827)
Supplement: Supplementary file 1 [file jcm-15-07827-s001.zip › Table_S5_Complete_case_analysis.pdf]

Table S5. Complete-case analysis restricted to the 39 patients who underwent molecular testing.

Model	n	Events (PFS / OS)	EPV (PFS / OS)	PFS HR (95% CI)	P	OS HR (95% CI)	P
Full cohort (primary model)	53	38 / 35	9.5 / 8.8	0.42 (0.19–0.95)	0.037	0.32 (0.12–0.85)	0.022
Tested patients (primary model structure)	39	28 / 25	7.0 / 6.3	0.51 (0.20–1.29)	0.155	0.32 (0.10–1.02)	0.055
Tested patients + MGMT adjustment	39	28 / 25	7.0 / 6.3	0.49 (0.20–1.19)	0.116	0.34 (0.13–0.87)	0.025
Tested patients (univariable)	39	28 / 25	—	0.50 (0.23–1.09)	0.082	0.48 (0.21–1.10)	0.083

EPV, events per variable; other abbreviations as in Table S1. Restricting the analysis to tested patients reduces the number of events from 38 to 28 (PFS) and from 35 to 25 (OS), lowering the EPV from 9.5 / 8.8 to 7.0 / 6.3; the loss of statistical significance for PFS in this subset is therefore a consequence of reduced power rather than disappearance of the effect. For overall survival the benefit remained statistically significant after additional adjustment for MGMT promoter methylation. The treatment effect could not be estimated meaningfully in the 14 untested patients (OS HR 5.77, 95% CI 0.33–102.5); with a confidence interval spanning three orders of magnitude this estimate carries no information and is reported only for completeness.
