# Supplementary material for: The Efficacy and Safety of Adding Teniposide to Temozolomide Plus Bevacizumab in Recurrent Grade 4 Glioma: A Real-World Exploratory Single-Center Study
Source: J Clin Med. 2026 Oct 10;15(20):7827. doi: 10.3390/jcm15207827 (PMC13705379; doi:10.3390/jcm15207827)
Supplement: Supplementary file 1 [file jcm-15-07827-s001.zip › Table_S6_Selection_bias_tested_vs_untested.pdf]

Table S6. Baseline characteristics of patients who underwent molecular testing versus those who did not (assessment of selection bias).

Characteristic	Tested (n = 39)	Not tested (n = 14)	SMD
Gross total resection at initial surgery	41.0%	14.3%	+0.626
Treatment: VM26 + TMZ + Bev	56.4%	28.6%	+0.587
Recurrence surgery before chemotherapy	23.1%	50.0%	−0.582
Age, years	52.6	60.5	−0.501
Male sex	43.6%	64.3%	−0.424
Body-mass index, kg/m^2	23.6	23.2	+0.101
Any comorbidity	46.2%	50.0%	−0.077
Multiple lesions at recurrence	30.8%	28.6%	+0.048
KPS ≥ 90	76.9%	78.6%	−0.040

SMD, standardised mean difference. Continuous variables are summarized as means because the presentation here is descriptive. Glioblastoma IDH-wildtype is not shown: patients who did not undergo testing cannot be assigned an IDH status, so this variable is structurally zero in the untested group and its SMD is an artefact rather than a real difference. Median PFS was 158 versus 174 days and median OS 247 versus 227 days; testing status was not associated with outcome on log-rank testing (P = 0.266). Patients who underwent testing were therefore younger, more often had gross total resection at initial surgery and were more often treated with the VM-26-containing regimen — a pattern consistent with selection towards a favorable-risk population — but the two groups did not differ in survival.
