# Supplementary material for: The Efficacy and Safety of Adding Teniposide to Temozolomide Plus Bevacizumab in Recurrent Grade 4 Glioma: A Real-World Exploratory Single-Center Study
Source: J Clin Med. 2026 Oct 10;15(20):7827. doi: 10.3390/jcm15207827 (PMC13705379; doi:10.3390/jcm15207827)
Supplement: Supplementary file 1 [file jcm-15-07827-s001.zip › Table_S7_Multicollinearity_diagnostics.pdf]

Table S7. Multicollinearity diagnostics among the molecular and clinical covariates.

Variable pair	Correlation coefficient (r)	VIF	Assessment
MGMT ~ CDKN2A/B	+0.858	6.21 / 5.45	Moderate
MGMT ~ TERT	+0.825	6.21 / 4.44	Moderate
TERT ~ CDKN2A/B	+0.792	4.44 / 5.45	Moderate
Glioblastoma IDH-wildtype ~ MGMT (negative)	−0.721	4.21 / 6.21	Moderate
Age ~ any comorbidity	+0.498	1.93 / 1.90	Acceptable

VIF, variance inflation factor. VIF > 5 indicates moderate and VIF > 10 severe collinearity. Because MGMT, TERT and CDKN2A/B were moderately collinear with one another, the primary model used the integrated diagnosis (glioblastoma, IDH-wildtype) rather than individual molecular markers; MGMT was examined only in the complete-case sensitivity analysis (Table S5).
