# Supplementary material for: The Efficacy and Safety of Adding Teniposide to Temozolomide Plus Bevacizumab in Recurrent Grade 4 Glioma: A Real-World Exploratory Single-Center Study
Source: J Clin Med. 2026 Oct 10;15(20):7827. doi: 10.3390/jcm15207827 (PMC13705379; doi:10.3390/jcm15207827)
Supplement: Supplementary file 1 [file jcm-15-07827-s001.zip › Table_S8_Survival_analyses_by_treatment_era.pdf]

Table S8. Survival analyses accounting for treatment era.

Analysis	Endpoint	n	Median, months (95% CI) † triplet / later era	Median, months (95% CI) † control / earlier era	HR (95% CI)	P
Era-matched comparison of the overlap-period cohorts (both groups treated 2023–2026) ‡	PFS	26 vs 15	7.8 (4.9–10.9)	6.3 (2.9–8.3)	0.62 (0.28–1.36)	0.228 §
	OS	26 vs 15	12.7 (8.4–15.4)	9.7 (5.5–10.3)	0.60 (0.25–1.45)	0.252 §
Era-matched comparison, adjusted for baseline covariates ¶	PFS	41	—	—	0.57 (0.22–1.46)	0.244
	OS	41	—	—	0.46 (0.15–1.39)	0.168
Cross-era comparison of the identical TMZ + Bev regimen (overlap vs non-overlap period) ‡	PFS	15 vs 12	6.3 (2.9–8.3)	4.7 (4.0–7.0)	0.76 (0.33–1.73)	0.507 §
	OS	15 vs 12	9.7 (5.5–10.3)	8.2 (6.8–9.7)	0.82 (0.34–1.95)	0.648 §
Pooled comparison with treatment era included as a covariate ‖	PFS	53	—	—	0.58 (0.26–1.29)	0.183
	OS	53	—	—	0.55 (0.23–1.32)	0.182

HR, hazard ratio; CI, confidence interval; PFS, progression-free survival; OS, overall survival; VM26, teniposide; TMZ, temozolomide; Bev, bevacizumab. All hazard ratios compare the VM-26-containing triplet with TMZ + bevacizumab; HR < 1 indicates benefit for the triplet or, in the cross-era comparison, for the later period. † Median survival was estimated by the Kaplan–Meier method and the 95% CI was derived from the Greenwood variance estimator with a log–log transformation; median survival times are expressed in months, calculated as days divided by 30.4375. ‡ In the era-matched comparison both groups were treated during the same calendar period (2023–2026), so the contrast estimates the effect of adding teniposide while holding treatment era constant; in the cross-era comparison both groups received the identical TMZ + Bev regimen, so the contrast quantifies the secular change in outcome over time. § Two-sided log-rank P value comparing the two groups. ¶ Adjusted for KPS ≥ 90, glioblastoma IDH-wildtype, re-resection at recurrence completed before chemotherapy and multiple lesions at recurrence; 41 of the 53 patients had complete data. The model is exploratory: 26 PFS and 23 OS events with five covariates correspond to approximately five events per variable. ‖ Treatment era was included as a binary covariate (overlap period 2023–2026 vs non-overlap period 2020–2022); because all patients treated with the triplet were treated during the overlap period, treatment and era are structurally collinear and this model is reported for completeness only. No baseline variable differed significantly between the two control eras (all P ≥ 0.25) or between the two concurrently treated groups (all P ≥ 0.30). All analyses are on an intention-to-treat basis and are not adjusted for multiplicity.
