# Supplementary material for: The Efficacy and Safety of Adding Teniposide to Temozolomide Plus Bevacizumab in Recurrent Grade 4 Glioma: A Real-World Exploratory Single-Center Study
Source: J Clin Med. 2026 Oct 10;15(20):7827. doi: 10.3390/jcm15207827 (PMC13705379; doi:10.3390/jcm15207827)
Supplement: Supplementary file 1 [file jcm-15-07827-s001.zip › Table_S9_Clinical_considerations_for_treatment_selection.pdf]

Table S9. Clinical considerations recorded at the time of treatment selection, by treatment group.

Patient	Treatment arm	Recorded clinical considerations for regimen selection
1	VM26 + TMZ + Bev	Good performance status; Progressive disease despite prior therapy; MGMT promoter methylation; Radiation necrosis
2	VM26 + TMZ + Bev	Good performance status; MGMT promoter methylation
3	VM26 + TMZ + Bev	Good performance status; Progressive disease despite prior therapy
4	VM26 + TMZ + Bev	Good performance status; Progressive disease despite prior therapy; Tumor considered difficult to resect; MGMT promoter methylation
5	VM26 + TMZ + Bev	Good performance status; Progressive disease despite prior therapy; Multiple lesions at recurrence
6	VM26 + TMZ + Bev	Good performance status; Progressive disease despite prior therapy; MGMT promoter methylation; Radiation necrosis
7	VM26 + TMZ + Bev	Good performance status; Progressive disease despite prior therapy; Tumor considered difficult to resect
8	VM26 + TMZ + Bev	Good performance status; Progressive disease despite prior therapy; Tumor considered difficult to resect; Multiple lesions at recurrence
9	VM26 + TMZ + Bev	Good performance status; Progressive disease despite prior therapy; Multiple lesions at recurrence; MGMT promoter methylation
10	VM26 + TMZ + Bev	Intermediate performance status; Tumor considered difficult to resect; Multiple lesions at recurrence
11	VM26 + TMZ + Bev	Good performance status; Tumor considered difficult to resect; Multiple lesions at recurrence
12	VM26 + TMZ + Bev	Good performance status; Tumor considered difficult to resect; MGMT promoter methylation
13	VM26 + TMZ + Bev	Good performance status
14	VM26 + TMZ + Bev	Good performance status; Tumor considered difficult to resect
15	VM26 + TMZ + Bev	Good performance status
16	VM26 + TMZ + Bev	Good performance status; Progressive disease despite prior therapy; Tumor considered difficult to resect; Multiple lesions at recurrence
17	VM26 + TMZ + Bev	Good performance status; Multiple lesions at recurrence
18	VM26 + TMZ + Bev	Good performance status; Tumor considered difficult to resect; Multiple lesions at recurrence
19	VM26 + TMZ + Bev	Good performance status; Tumor considered difficult to resect; Multiple lesions at recurrence
20	VM26 + TMZ + Bev	Good performance status; Progressive disease despite prior therapy
21	VM26 + TMZ + Bev	Good performance status
22	VM26 + TMZ + Bev	Good performance status; Progressive disease despite prior therapy
23	VM26 + TMZ + Bev	Good performance status; Tumor considered difficult to resect
24	VM26 + TMZ + Bev	Poor performance status; Progressive disease despite prior therapy; Tumor considered difficult to resect; MGMT promoter methylation; Radiation necrosis

25	VM26 + TMZ + Bev	Good performance status; Progressive disease despite prior therapy; Multiple lesions at recurrence; MGMT promoter methylation
26	VM26 + TMZ + Bev	Good performance status
27	TMZ + Bev	Good performance status
28	TMZ + Bev	Good performance status; Tumor considered difficult to resect; MGMT promoter methylation
29	TMZ + Bev	Good performance status; Multiple lesions at recurrence
30	TMZ + Bev	Good performance status
31	TMZ + Bev	Good performance status; Tumor considered difficult to resect; Multiple lesions at recurrence
32	TMZ + Bev	Good performance status
33	TMZ + Bev	Good performance status
34	TMZ + Bev	Good performance status; Tumor considered difficult to resect
35	TMZ + Bev	Good performance status; Progressive disease despite prior therapy
36	TMZ + Bev	Good performance status; Progressive disease despite prior therapy
37	TMZ + Bev	Good performance status; Progressive disease despite prior therapy
38	TMZ + Bev	Good performance status; Progressive disease despite prior therapy
39	TMZ + Bev	Intermediate performance status; Progressive disease despite prior therapy; Tumor considered difficult to resect; Multiple lesions at recurrence; MGMT promoter methylation
40	TMZ + Bev	Good performance status; Progressive disease despite prior therapy; Multiple lesions at recurrence
41	TMZ + Bev	Good performance status; Tumor considered difficult to resect; Multiple lesions at recurrence; MGMT promoter methylation
42	TMZ + Bev	Good performance status; MGMT promoter methylation
43	TMZ + Bev	Intermediate performance status; Multiple lesions at recurrence
44	TMZ + Bev	Good performance status; Progressive disease despite prior therapy
45	TMZ + Bev	Good performance status; Progressive disease despite prior therapy
46	TMZ + Bev	Intermediate performance status; Progressive disease despite prior therapy; Radiation necrosis
47	TMZ + Bev	Good performance status; Progressive disease despite prior therapy
48	TMZ + Bev	Good performance status; Progressive disease despite prior therapy; MGMT promoter methylation
49	TMZ + Bev	Good performance status; Progressive disease despite prior therapy
50	TMZ + Bev	Good performance status; Progressive disease despite prior therapy
51	TMZ + Bev	Good performance status; Tumor considered difficult to resect
52	TMZ + Bev	Good performance status; Progressive disease despite prior therapy
53	TMZ + Bev	Good performance status; Progressive disease despite prior therapy

VM26, teniposide; TMZ, temozolomide; Bev, bevacizumab; MGMT, O^6-methylguanine-DNA methyltransferase. The considerations listed were recorded in the medical record at the time the regimen was selected and were abstracted retrospectively for every patient; several patients had more than one recorded consideration, and the number per patient ranged from one to five. Recorded considerations in the VM26 + TMZ + Bev group (n = 26) versus the TMZ + Bev control group (n = 27): good performance status 24 vs 24; progressive disease despite prior therapy 13 vs 15; tumour considered difficult to resect 12 (46.2%) vs 6 (22.2%); multiple lesions at recurrence 10 (38.5%) vs 6 (22.2%); MGMT promoter methylation 8 (30.8%) vs 5 (18.5%); radiation necrosis 3 (11.5%) vs 1 (3.7%); intermediate performance status 1 vs 3; poor performance status 1 vs 0. Patients are numbered consecutively within each group; no identifying information is included.
